# Purkinje cell hyperexcitability and depressive-like behavior in mice lacking erg3 (ether-à-go-go–related gene) K^+^ channel subunits

**DOI:** 10.1126/sciadv.adn6836

**Published:** 2024-10-04

**Authors:** Jürgen R. Schwarz, Sandra Freitag, Yvonne Pechmann, Irm Hermans-Borgmeyer, Wolfgang Wagner, Sönke Hornig, Matthias Kneussel

**Affiliations:** ^1^ Institute of Molecular Neurogenetics, Center for Molecular Neurobiology Hamburg (ZMNH), University Medical Center Hamburg-Eppendorf (UKE), Hamburg, Germany.; ^2^ Core Facility Transgenic Animals, Center for Molecular Neurobiology Hamburg (ZMNH), University Medical Center Hamburg-Eppendorf (UKE), Hamburg, Germany.

## Abstract

Potassium channels stabilize the resting potential and neuronal excitability. Among them, erg (ether-à-go-go–related gene) K^+^ channels represent a subfamily of voltage-gated channels, consisting of erg1, erg2, and erg3 subunits; however, their subunit-specific neuronal functions in vivo are barely understood. To find erg3- and erg1-mediated functions, we generated global *Kcnh7* (*erg3*) and conditional *Kcnh2* (*erg1*) knockout mice. We found that erg3 channels stabilize the resting potential and dampen spontaneous activity in cerebellar Purkinje cells (PCs) and hippocampal CA1 neurons, whereas erg1 channels have suprathreshold functions. Lack of erg3 subunits induced hyperexcitability with increased action potential firing in PCs, but not in CA1 neurons. Notably, erg3 depletion caused depressive-like behavior with reduced locomotor activity, strongly decreased digging behavior, and shorter latencies to fall off a rotating wheel, while learning and memory remained unchanged. Our data show that erg K^+^ channels containing erg3 subunits mediate a neuronal subthreshold K^+^ current that plays important roles in the regulation of locomotor behavior in vivo.

## Teaser

Mice lacking erg3 K channels exhibit hyperexcitability of Purkinje cells and depressive-like behavior.

## INTRODUCTION

The erg (ether-à-go-go–related gene) K^+^ channel family consists of tetrameric ion channels formed by homo- and/or heteromeric combinations of three individual subunits erg1, erg2, and erg3 (also known as Kv11.1, Kv11.2, and Kv11.3), which have been functionally characterized in vitro (*1*–*3*). In situ hybridization and immunohistochemical studies revealed that all three erg subunits are expressed throughout the rodent brain, with distinct and overlapping expression patterns of erg1 and erg3 subunits but relatively poor expression of erg2 subunits (*4*–*7*). Although it has been shown that erg K^+^ channels are effective modulators of neuronal excitability (*8*, *9*), the specific functions of individual erg subunits with respect to excitability properties of single neurons and higher order brain functions are not yet known.

Erg channels are characterized by “inverted” gating kinetics, i.e., they activate and deactivate slowly but inactivate and recover from inactivation with fast kinetics (*10*). In the human heart erg1 (HERG), currents (together with KCNQ1 currents) control action potential repolarization, i.e., their inhibition prolongs the plateau phase of the ventricular cardiac action potential. This prolongation increases the likelihood of early after-depolarizations, which are at risk to induce heart arrhythmia and occasionally sudden death (LQT2 syndrome) (*10*). Malfunction of HERG can be caused by mutations in *Kcnh2*, the human gene encoding erg1a, but is more often induced by a broad range of drugs (HERG channel blockers) prescribed to treat cardiac, mental, or other diseases.

In contrast to the well-defined cardiac function of HERG, specific neuronal functions of each of the three erg channels are only beginning to be identified. In heterologous expression systems, individual erg channel subunit combinations exhibit distinct potential- and time-dependent biophysical properties: Homomeric erg3 channels activate near the resting potential of about −60 mV, whereas homomeric erg1 or erg2 channels activate at more depolarized potentials. Compared to erg3 channels, their activation curves are shifted by 20 or 40 mV, respectively, to more positive membrane potentials (*2*, *11*). In addition, erg3 channels activate faster and generate larger sustained currents near the threshold potential, as compared to homomeric erg1 or erg2 channels. These distinct biophysical properties suggest that the three types of erg channels control different neuronal functions. Accordingly, erg3-containing channels are believed to mediate subthreshold K^+^ currents with large window currents close to the resting potential, whereas erg1- or erg2-containing channels activate at more positive membrane potentials (*2*, *9*, *12*).

Since erg2 mRNA is poorly expressed in the brain, neuronal erg K^+^ currents are either subthreshold or suprathreshold currents depending on whether homo- and/or heteromeric channels are composed of predominantly erg3 and/or erg1 subunits. In several brain regions, erg channels have been shown to mediate subthreshold currents, i.e., they stabilize the resting potential and dampen spontaneous firing of action potentials in subthalamic nucleus neurons (*13*), mitral cells of the olfactory bulb (*14*), cerebellar Purkinje cells (PCs) (*15*, *16*), and hippocampal CA1 neurons (*17*). In these examples, erg channels containing erg3 subunits may play an important role. In contrast, neuronal erg1 subunits may contribute to erg K^+^ channels that activate at more positive membrane potentials; for instance, they could be involved in the accommodation of repetitive action potential firing in sensory neurons (*18*) or in controlling the duration of long-lasting action potentials of dopaminergic neurons (*19*).

In almost all reports on the neuronal function of erg-type channels, sulfonanilides such as E-4031 or WAY-123,398 have been used to block erg channels. Sulfonanilides inhibit all erg channels with similar selectivity and potency (*2*) and are therefore excellent tools for isolating erg-mediated K^+^ channel currents from other endogenous ion channel currents (*16*). However, erg subunit–specific pharmacological blockers are missing; therefore, pharmacological isolation of individual erg K^+^ channel subtypes is currently not possible. Alternatively, transgenic mice have recently been used to identify neuronal erg channels. For example, erg1-containing channels have been identified in inner hair cells of the mouse cochlea, which mediate the K^+^ current involved in precise encoding of sound (*20*). Erg3 channels have also been detected in inferior olivary neurons that mediate the K^+^ current that controls resonance and membrane oscillations near the resting potential (*21*).

Here, we used mouse genetics to investigate the contribution of erg3 subunits to neuronal excitability and behavior in vivo. On the basis of the dominant role of erg3 subunits in cerebellar PCs (*15*) and hippocampal CA1 neurons (*17*), we compared these two cell types at the level of individual neurons. We found that cerebellar PCs lacking erg3 subunits are hyperexcitable, whereas the excitability of hippocampal CA1 neurons lacking erg3 subunits remains unchanged. Accordingly, at the behavioral level, we found that loss of erg3 subunits induces a depressive-like locomotor behavior rather than defects in learning and memory. Our data indicate that erg K^+^ channels containing erg3 subunits play a critical role in regulating neuronal excitability and higher brain function.

## RESULTS

### Generation of two mouse lines to analyze the function of neuronal erg channels

**Fig. 1.**
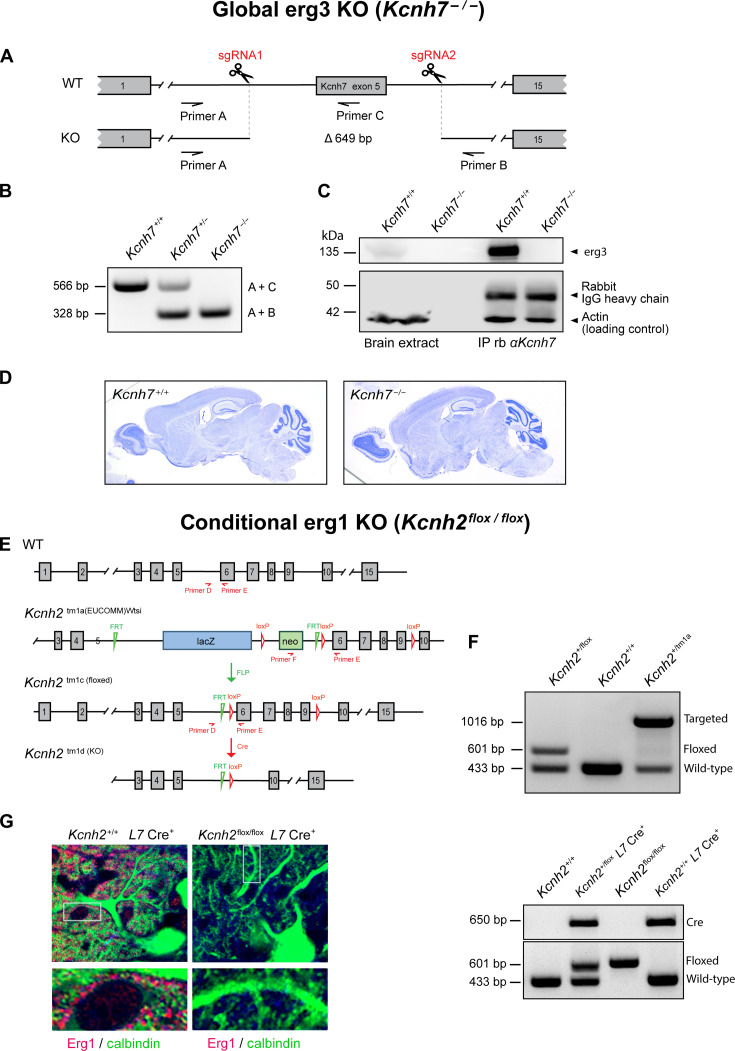
Generation of global *Kcnh7* KO and conditional PC-specific *Kcnh2* KO. (**A**) *Kcnh7* locus in WT or *Kcnh7*-deficient (KO) mice. Boxes, exons; horizontal lines, introns. Arrows, location of primers used for genotyping. Scissors, sgRNA target sequence. A deletion of 649 base pairs (bp) leads to a premature stop codon after 316 amino acids. (**B**) PCR with genotypes *Kcnh7*^+/+^, *Kcnh7*^+/−^, and *Kcnh7*^−/−^. WT, primer A + C 566 bp; KO, 328 bp. The predicted WT band A + B of 977 bp is not amplified under PCR conditions used. (**C**) Absence of erg3 protein in brain extracts of homozygous *Kcnh7^em2Hhtg^* mice. Lanes 1 and 2, Western blot analysis using brain extracts from 12- to 16 week-old WT (*Kcnh7^+/+^*) and homozygous KO (*Kcnh7^−/−^*) mice. Lanes 3 and 4, erg3 is precipitated with a *Kcnh7*(erg3)-specific antibody from brain lysates of WT (*Kcnh7^+/+^*) but not from homozygous KO (*Kcnh7^−/−^*) mice. Actin detection, loading control. IgG, immunoprecipitation control. (**D**) Nissl staining of sagittal brain sections from adult WT (*Kcnh7^+/+^*) and *Kcnh7^em2Hhtg^* KO (*Kcnh7^−/−^*) mice. (**E**) *Kcnh2* KO mouse generation. Mice homozygous for the floxed allele of *Kcnh2* (*Kcnh2^flox/flox^*) and heterozygous for *L7* Cre (*L7* Cre+) were crossed to obtain PC-specific *Kcnh2* KOs. (**F**) Top, representative PCR showing genotypes *Kcnh2*^+/flox^, *Kcnh2*^+/+^, and *Kcnh2*^+/tm1a^. Floxed allele, primers D + E 601 bp; WT allele, primers D + E 433 bp; targeted allele, primers E + F 1016 bp. Bottom, PCR of genotypes *Kcnh2*^+/+^ *L7* Cre neg., *Kcnh2*^+/flox^ *L7* Cre pos., *Kcnh2* ^flox/flox^ *L7* Cre neg., and *Kcnh2*^+/+^ *L7* Cre pos.. Floxed allele, primers D + E 601 bp; WT allele, primers D + E 433 bp; L7-Cre primers G + H (see Table 1): 650 bp. (**G**) Sagittal cerebellar sections from adult control (*Kcnh2^+/+^ L7Cre pos.*) and PC-specific KO (*Kcnh2^flox/flox^* L7Cre pos.) mice immuno-fluorescently labeled with antibodies against Erg1 and calbindin-D-28 K (PC marker).

Previous results suggest that erg currents recorded in PCs are mediated by heteromeric erg1/erg3-containing K^+^ channels (*15*). To identify the currents mediated by erg1 and erg3 subunits, we used two different knock-out (KO) strategies. First, we generated a mouse line with a global KO of *Kcnh7*, the gene encoding erg3 subunits. For this purpose, we deleted exon 5 of the *Kcnh7* gene using the CRISPR-Cas9 technique (Fig. 1, A to C) (*22*). Nissl staining of tissue slices from homozygous mutants (referred to as *Kcnh7*-KO or *Kcnh7*^−/−^ mice) revealed no gross anatomical abnormalities of the brain regarding foliation or organization of cellular layers in the cortex, hippocampus, and cerebellum (Fig. 1D), indicating that erg3 depletion neither disturbs major brain development nor induces neurodegeneration. Homozygous *Kcnh7*-KO mice were viable and showed no obvious weight or size deficits, compared with wild-type littermate controls (referred to as WT or *Kcnh7*^+/+^). We therefore used this mouse line to investigate the consequence of erg3 subunit depletion with respect to neuronal excitability and behavior. Since a global KO of *Kcnh2* is lethal (*23*), we further generated a mouse line containing floxed exons 6 to 9 of *Kcnh2*, the gene encoding erg1a subunits. Following crossbreeding with FLP deleter and *L7* (Pcp2)–Cre lines (*24*, *25*), we specifically inhibited *Kcnh2* gene expression in PCs (referred to as conditional *Kcnh2*-KO, (Fig. 1, E and F), as proven by immunohistochemistry (Fig. 1G).

**Table 1.** Primers used for genotyping.

A	*Kcnh7*-F	GTA GAG ACT CCG TGG ATC ATT TCA TAT AGG TA
B	*Kcnh7*-rev	CCA AGT ATG ATG AAT AGC TCA GTA ATT ATT TCA GAG CA
C	YP_*KCNH7*_ex6-as	GTT TGA ATC TGA TGT GGA TCC CAG C
D	YP-*KCNH2*ex5-s	GTG GAG AGA AGC CTG AGA AAG
E	YP-*KCNH2*-UTR6-as	GAC ATA CCA GCT GAC AGT AC
F	NEO-F	CGT TGG CTA CCC GTG ATA TT
G	CRE-F	GGC AGT AAA AAC TAT CCA GC
H	CRE-R	TCC GGT ATT GAA ACT CCA GC

### Erg current of PCs consists of erg1 and erg3 components, whereas erg current of CA1 neurons is mediated by erg3-containing channels

We first compared two prominent neuronal cell types with distinct functional properties in the brain, namely cerebellar PCs and hippocampal CA1 neurons, as erg3 currents are believed to be functionally dominant in both cell types (*15*, *17*). Patch-clamp experiments were performed in PCs to record membrane currents in acute brain slices from 6- to 14-day-old mice. At this age, membrane potential control by the voltage clamp is sufficient as the dendritic tree of PCs is not yet developed (*26*). Since amplitudes of these neuronal erg K^+^ currents were small, they were increased by using elevated K^+^ concentrations in the external solution (40 mM KCl), and removal of Ca^2+^ from this solution avoided activation of Ca^2+^-dependent K^+^ channels (*1*, *12*, *15*, *21*, *27*). Erg-mediated K^+^ channel currents from CA1 neurons were recorded in slices obtained from slightly older animals (2 to 4 weeks).

**Fig. 2.**
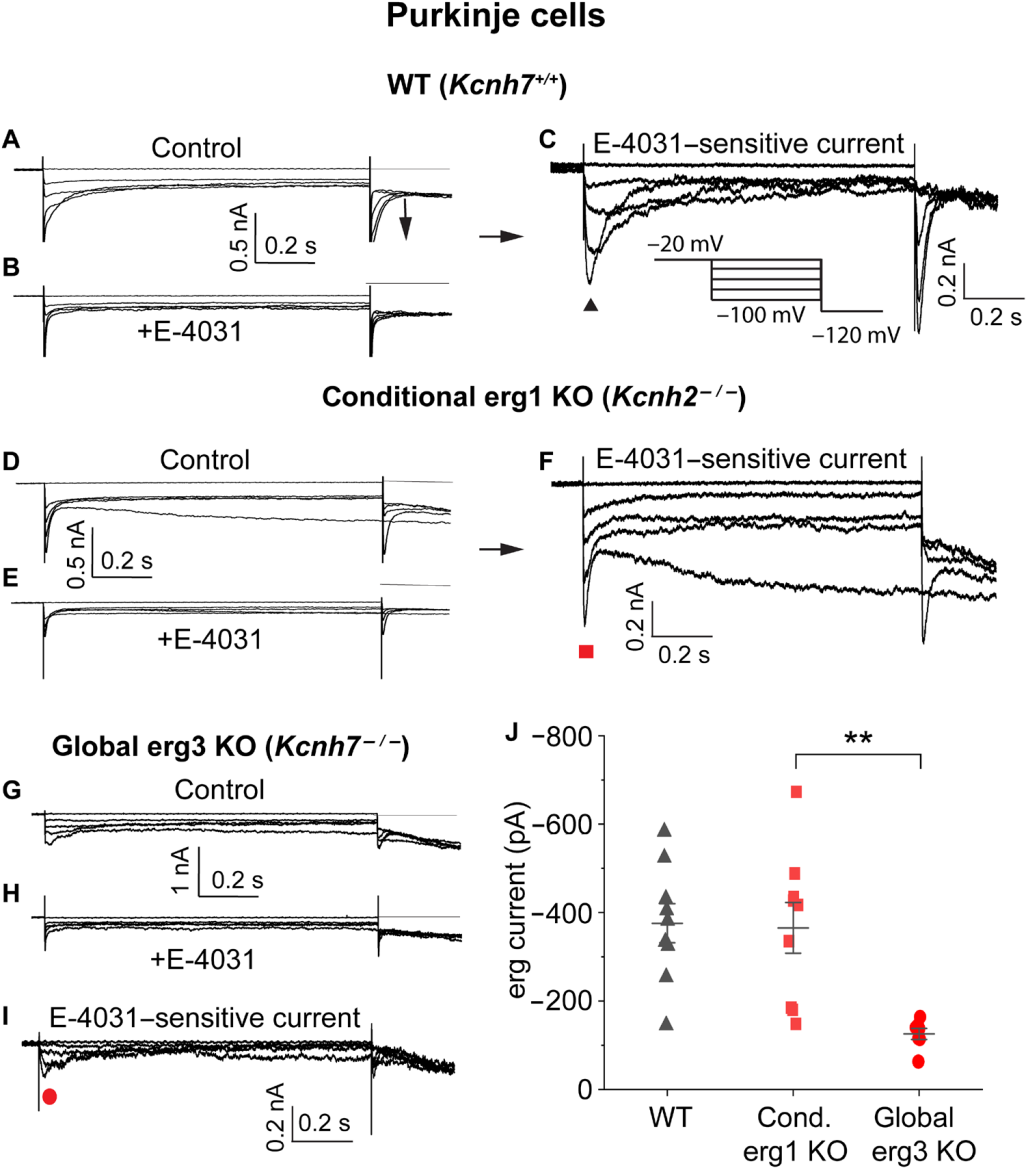
Dissection of PC erg current into two components. Dissection of the erg current of PCs into two components using two different KO mice. (**A** to **C**) Isolation of E-4031–sensitive erg K^+^ current of a PC from a *Kcnh7*^+/+^ (WT) mouse. Membrane currents recorded from a PC in an acute cerebellar slice obtained from a WT mouse (age, 6 days) in the absence [(A) control] and in the presence of E-4031 [(B) +E-4031]. Recordings were performed in artificial cerebrospinal fluid (ACSF) containing 40 mM KCl without Ca^2+^ using the pulse protocol shown in (C). Subtraction of currents in (B) from those in (A) yielded the E-4031–sensitive erg currents (C). (**D** to **F**) Membrane currents recorded from a PC of a conditional *Kcnh2*-KO mouse in the absence (D) and presence of E-4031 (E). Subtraction of currents in (E) from those in (D) yielded the E-4031–sensitive current (F). (**G** to **I**) Erg currents recorded from a PC of a *Kcnh7^−/−^* mouse (age, 7 days) in an acute cerebellar slice. Membrane currents recorded in a solution as in the WT control (G). Application of E-4031 reduced membrane currents (H). E-4031–sensitive currents (I) obtained by subtraction of membrane currents shown in (H) from those in (G). Peak current amplitudes were evaluated as indicated by the symbols. (**J**) Graph plotting erg current amplitudes from PCs of WT, conditional erg1 KO and global erg3 KO mice.

In these experiments, we used a deactivation protocol (see pulse protocol in Fig. 2C, inset) and exploited the specific sensitivity of erg K^+^ channels to the channel blocker E-4031. From a holding potential of −20 mV, hyperpolarizing potentials (with increasing steps of 20 mV) elicited transient inward currents generated by fast recovery from erg K^+^ channel inactivation and subsequent slow deactivation. In PCs from WT mice, membrane currents were reduced by E-4031 (10 μM) (Fig. 2, A and B) and subtraction of the currents recorded in the presence of E-4031 from the control currents yielded the E-4031–sensitive currents (Fig. 2C). Since deactivation proceeds faster at more negative membrane potentials, currents elicited with a −100-mV pulse led to a characteristic crossing-over of erg K^+^ current traces (*15*). With the final potential step to −120 mV erg, K^+^ channel tail currents were recorded. Under these conditions, the mean peak amplitude of the E-4031–sensitive current elicited with a −100-mV potential step was *I*_erg_ = −376 ± 44 pA (*n* = 9) (Fig. 2J).

E-4031–sensitive currents recorded in conditional *Kcnh2* KO PCs, lacking erg1subunits, revealed current amplitudes of a similar magnitude as WT PCs (Fig. 2, F and J; *I*_erg_ = −365 ± 57 pA, *n* = 9), suggesting that erg3 is the main subunit forming erg K^+^ channels in this cell type. The functional significance of this erg current was demonstrated by on-cell recordings: The firing of spontaneous activity increased from 33 ± 4.7 to 52 ± 8.4 Hz (*n* = 20; *P* = 0.0018) after application of E-4031. Correspondingly, patch-clamp recordings from PCs of mice lacking erg3-type K^+^ channel subunits (*Kcnh7*^−/−^, referred to as global KO) exhibited an E-4031–sensitive current with relatively small amplitudes (*I*_erg_ = −125 ± 12 pA, *n* = 7; Fig. 2, I and J).

**Fig. 3.**
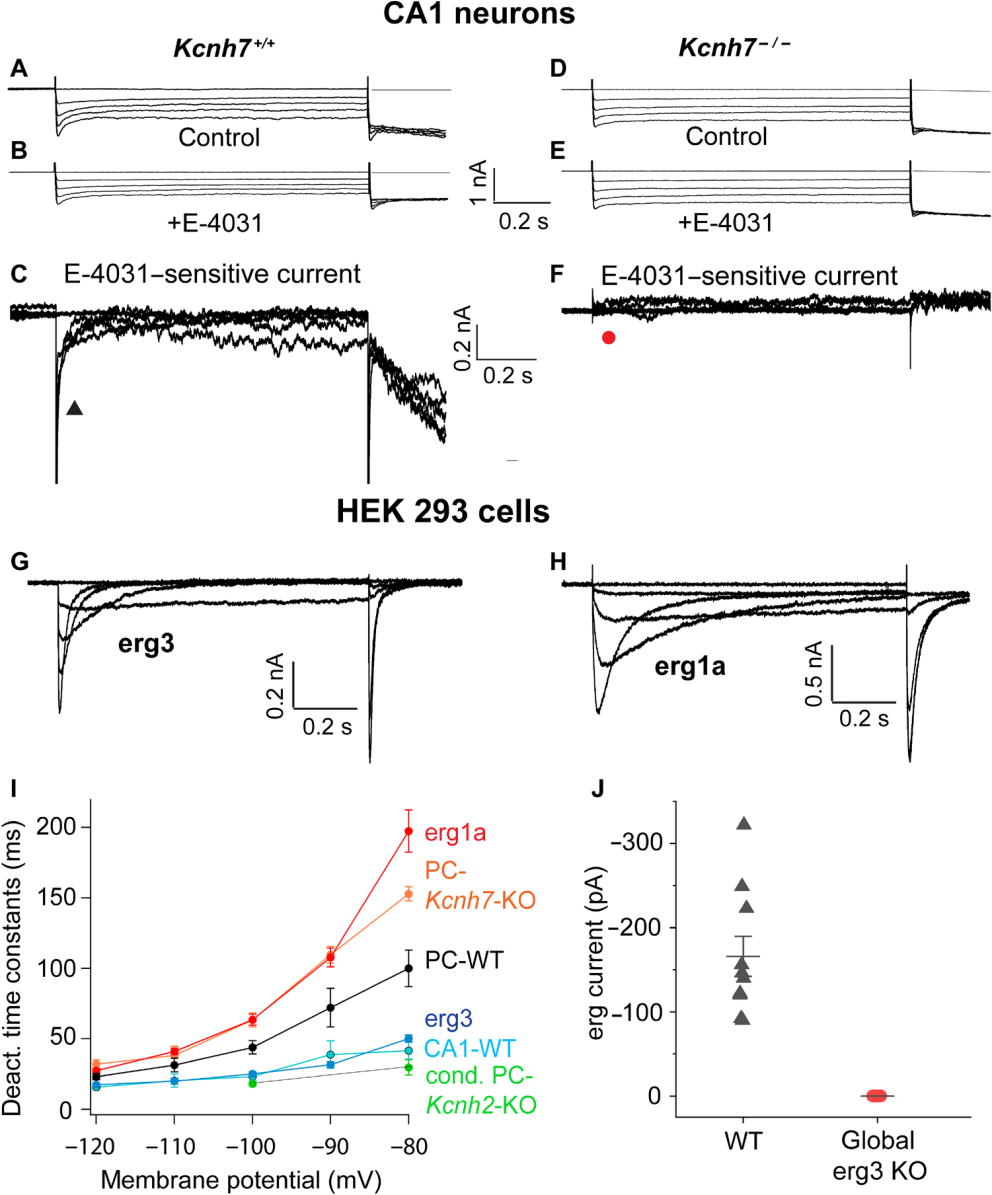
The erg current of CA1 neurons is mediated only by erg3 channels. (**A** to **C**) Membrane currents recorded from a CA1 neuron in an acute cerebellar slice obtained from a WT mouse (age, 14 days) in the absence [(A) control] and presence of E-4031 [(B) +E-4031]. Recordings were performed in ACSF containing 40 mM KCl without Ca^2+^. The same pulse program used as in PCs (see Fig. 2C). (C) E-4031–sensitive currents obtained by subtraction of currents shown in (B) from those in (A). (**D** to **F**) Membrane currents recorded from a CA1 neuron of a *Kcnh7*-KO mouse (age, 12 days) in an acute hippocampal slice. (D) Membrane currents recorded in an extracellular solution in the absence (D) and presence of E-4031 (E). (F) No E-4031–sensitive current detectable by subtraction of membrane currents in (E) from those in (D). (**G** and **H**) Membrane currents mediated by homomeric erg3 (G) and erg1a channels (H) stably expressed in HEK293 cells. Pulse protocol, see inset of Fig. 2C. Extracellular recording solution, Ringer’s solution containing 40 mM KCl. (**I**) Graph plotting deactivation time constants of erg1a and erg3 currents, erg currents recorded in PCs of WT, *Kcnh7*-KO mice, and conditional *Kcnh2* mice, as well as of CA1 neurons of WT mice against membrane potential. Mean values (see table S1) connected by straight lines. (**J**) Graph plotting erg current amplitudes recorded from CA1 neurons of WT and global erg3 KO mice.

Similar voltage-clamp experiments were performed in WT (*Kcnh7*^+/+^) CA1 neurons. In these neurons, deactivation of the E-4031–sensitive current proceeded with fast kinetics (Fig. 3, A to C). Unexpectedly, CA1 neurons of *Kcnh7*^−/−^ mice did not contain any remaining erg-type K^+^ channel current (Fig. 3, D to F). The mean erg K^+^ current amplitude (E-4031–sensitive current) elicited by the −100 mV potential step in WT CA1 neurons was found to be relatively small, amounting to *I*_erg_ = −166 ± 22 pA (*n* = 10) (Fig. 3J). These results supported the previous assumption that erg3 subunits play an important role in CA1 neurons (*17*).

To identify the erg current components recorded in PCs and CA1 neurons of WT and the different KO mice, we compared the deactivation time constants of native erg K^+^ currents with those of currents mediated by homomeric erg1a or erg3 channels expressed in human embryonic kidney (HEK) 293 cells (*11*, *12*). Erg3 currents (Fig. 3G) deactivate much faster than erg1a currents (Fig. 3H). Their deactivation time constants were almost identical to those of WT CA1 neurons, while the deactivation time constants of E-4031–sensitive currents of *Kcnh7*^−/−^ PCs were similar to those of erg1a-containing channels (Fig. 3I). The deactivation time constants determined in erg currents of WT PCs were intermediate between the time constants of erg1a- and erg3-mediated currents (Fig. 3I and table S1). These data suggested that E-4031–sensitive currents recorded in *Kcnh7*^−/−^ PCs are mediated by erg1-containing channels, whereas erg currents of WT CA1 neurons are mediated by erg3-containing channels (Fig. 3I). Last, the deactivation time constants of E-4031–sensitive currents recorded in erg1-depleted PCs from conditional *Kcnh2*-KO mice were similar to those of erg3 currents. Thus, we conclude that erg K^+^ currents of PCs consist of two components, erg1 and erg3, whereas erg currents of CA1 neurons are mediated by channels consisting of erg3 subunits only.

**Fig. 4.**
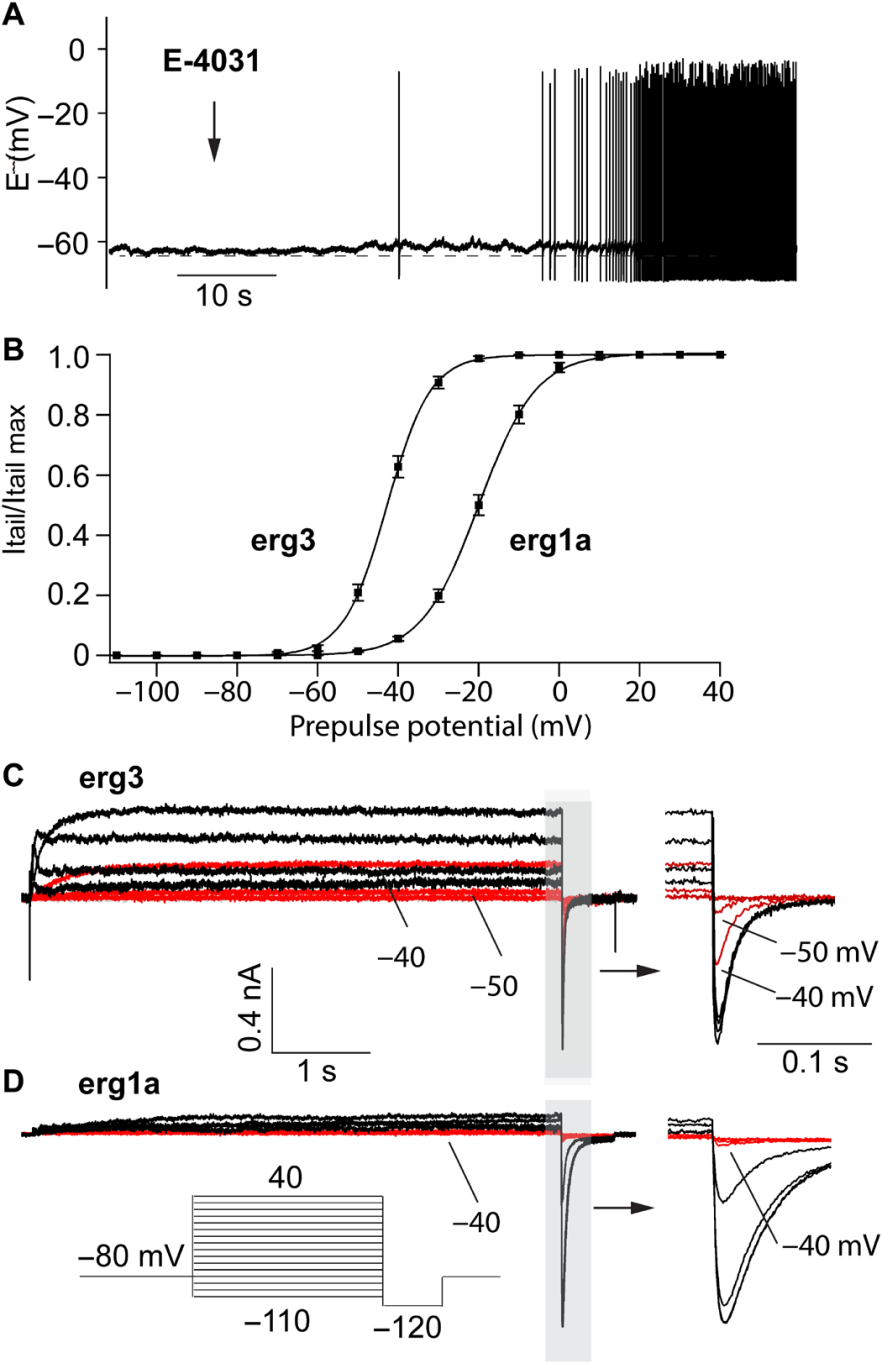
Erg3 channels mediate a subthreshold current and are involved in the maintenance of the resting potential. (**A**) Representative recording of the resting membrane potential of a WT PC (age, 3 weeks). Application of E-4031 depolarized the neuron and initiated irregular tonic firing of action potentials. Extracellular solution, standard ACSF, 37°C. (**B** to **D**) Membrane currents and activation curves of erg1a and erg3 channels (means ± SEM) determined in HEK293 cells expressing either erg1a or erg3 channels. Activation curves of erg1a and erg3 channels were determined from tail current amplitudes elicited with potential steps to −120 mV preceded by potentials between −110 and 40 mV in steps of 10 mV [pulse protocol in (D)]. A Boltzmann equation was fitted to the normalized mean values of the tail current amplitudes of erg1a (E_0.5_ = −20.0 mV; slope, 7.1 mV) and erg3 (E_0.5_ = −42.8 mV; slope, 5.4 mV) and plotted in the graph. Temperature, 21° to 23°C. (C) Erg3 current traces recorded from a holding potential of −80 mV with potential steps to −70, −60, −50, and −40 mV (red traces) and to −20, 0, 20, and 40 mV (black traces). Membrane currents were recorded in the absence and in the presence of E-4031. Both families of membrane currents were subtracted to obtain E-4031–sensitive currents. The last part of currents presented at an enlarged timescale. (D) Erg1a currents elicited with the same potential steps as in (C). The last part of currents presented at an enlarged timescale.

In a silent PC of a WT adolescent mouse, application of E-4031 (10 μM) depolarized the resting membrane potential by about 2 mV and induced spontaneous irregular firing of action potentials (Fig. 4A). To investigate whether erg3- or erg1a-containing K^+^ channels mediate this depolarization, we measured their membrane potential dependence of current activation in normal Ringer’s solution after heterologous expression in HEK293 cells (Fig. 4, B to D). The amplitudes of erg tail currents elicited with a potential step to −120 mV, preceded by potentials between −110 and 40 mV, were evaluated, normalized, plotted against prepulse potentials, and fitted to the Boltzmann equation (Fig. 4B). Characteristically, the activation curve of erg1a-type K^+^ channels was shifted by about 20 mV to more positive membrane potentials, compared to erg3-type K^+^ channels (*1*, *2*). A small fraction of homomeric erg3 channels is activated at the resting potential, thereby contributing to its maintenance (Fig. 4B). In addition, compared to erg1a-mediated currents, erg3-mediated currents activate faster and exhibit larger amplitudes within the subthreshold membrane potential range (Fig. 4, C and D). These data show that erg3-containing K^+^ channels exhibit more pronounced subthreshold properties than erg1-containing K^+^ channels.

### PCs from *Kcnh7*-KO mice lacking erg3 subunits are hyperexcitable

**Fig. 5.**
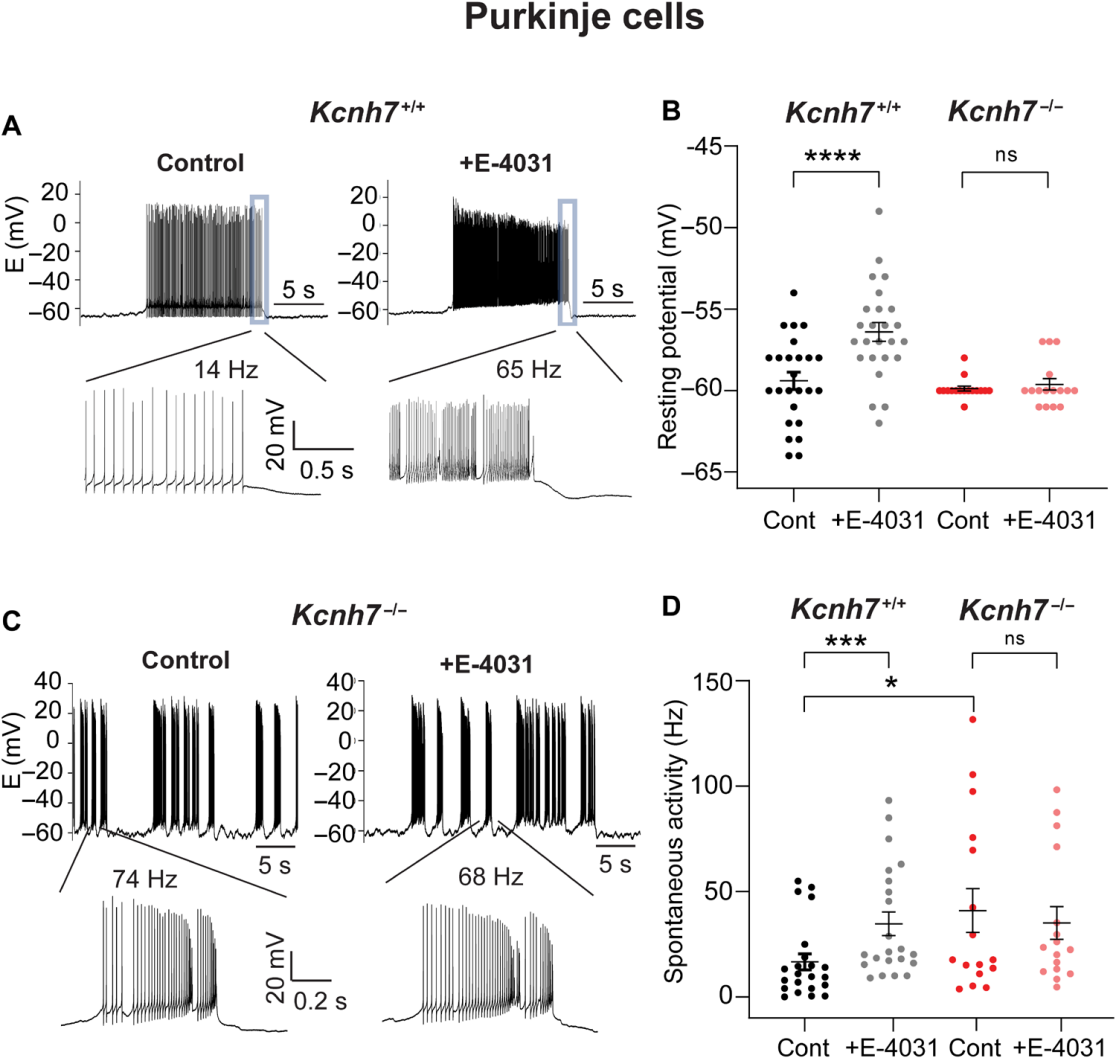
PCs lacking erg3 channel subunits (*Kcnh7*-KO) are hyperexcitable. Recordings of resting potential and spontaneous activity of PCs. (**A**) Spontaneous burst activity of a PC from a WT mouse (age, 4 weeks) recorded at 36°C in the absence (left, control) and in the presence of E-4031 (right, +E-4031). End of bursts (boxed) shown below on an expanded timescale. In the neuron shown the firing frequency increased from 14 to 65 Hz. (**B**) Plot of PC resting potentials of *Kcnh7*^+/+^ mice before (control, black) and in the presence of E-4031 (+E-4031, gray; *n* = 25, data from 11 mice; age, 3 to 8 weeks), as well as of PCs of *Kcnh7^−/−^* mice before (control, red) and in the presence of E-4031 (+E-4031, pink; *n* = 16; data from 6 mice). Paired *t* test used to calculate statistical significance. (**C**) PCs of *Kcnh7^−/−^* mice exhibited sustained hyperexcitability. Irregular spontaneous burst activity of a PC of a *Kcnh7^−/−^* mouse (age, 3 weeks) in the absence (left, control) and in the presence of E-4031 (right, +E-4031). Bottom, part of burst activity in the absence and in the presence of E-4031 (as indicated) shown on an expanded timescale. E-4031 did not significantly change burst action potential frequency. Temperature, 36°C. (**D**) Graph of spontaneous activity of PCs from *Kcnh7*^+/+^ mice in the absence (control, black; *n* = 22; data from 14 mice; age, 3 to 8 weeks) and in the presence of E-4031 (+E-4031, gray), as well as of PCs from *Kcnh7^−/−^* mice in the absence (control, red; *n* = 16; data from 6 mice) and in the presence of E-4031 (+E-4031, pink). Paired *t* test used to calculate statistical significance. Mann-Whitney test used to calculate significance between WT control and *Kcnh7*-KO control. ns, not significant. *P < 0.05, ***P < 0.001, ****P < 0.0001.

PCs of WT mice either spontaneously fired action potentials with low frequency or—more often—showed irregular tonic or burst activity. To investigate how erg3 subunit depletion affects neuronal excitability and behavior, we performed current-clamp experiments in PCs and CA1 neurons close to “in vivo” conditions, i.e., at near body temperature (33° to 36°C) and in the absence of synaptic blockers. Previous work in PCs had been done in the presence of synaptic blockers to remove any contribution of synaptic potentials to excitability. This work showed that blockage of erg channels with WAY-123,398 (*16*) or E-4031 (*15*) increased neuronal excitability and that this increase was presumably due to a change in the intrinsic membrane properties of PCs. Under in vivo conditions, we found that the resting potential of PCs was close to −60 mV (−59.4 ± 0.6 mV, *n* = 25; Fig. 5B, black). Application of E-4031 (10 μM) depolarized PCs by 2 to 5 mV (−56.4 ± 0.6 mV, *P* < 0.00001; Fig. 5B, gray) accompanied by either an initiation of action potentials (Fig. 4A) or an increase in the firing rate of spontaneous activity (Fig. 5A) from 16.7 ± 3.8 to 34.7 ± 5.6 Hz (*n* = 22; *P* = <0.0001; Fig. 5D. black gray). In PCs of *Kcnh7*-KO mice lacking erg3 subunits, the resting potential was initially depolarized (−57.8 ± 0.4 mV, *n* = 16) and significantly different (*P* < 0.05) from the resting potential of WT PCs. For better comparison, we adjusted the resting potential to similar values as in WT littermates (−59.9 ± 0.2 mV) by applying depolarizing or hyperpolarizing currents (Fig. 5B, red versus black). The resting potential remained unaltered (−59.6 ± 0.4 mV; Fig. 5B, pink) after application of E-4031 (10 μM), suggesting that erg3-containing K^+^ channels are critical in maintaining the resting potential in PCs. PCs from *Kcnh7*-KO mice exhibited significantly higher spontaneous resting activity, as compared to WT-PCs (41.0 ± 10.9 Hz; Fig. 5, C and D, red), their excitability was similar to PCs from WT mice recorded in the presence of E-4031. These values did not change in the presence of E-4031 (35.1 ± 8.1 Hz; *P* = 0.34; Fig. 5D, pink). We also investigated repetitive firing of PCs elicited by depolarizing potential steps. We found that E-4031 increased repetitive activity of WT-PCs elicited with low stimuli (fig. S1, A and C), whereas PCs from *Kcnh7*-KO mice had already higher control repetitive activity, which did not increase further after application of E-4031 (fig. S1, B and D). This increase in spontaneous and repetitive activity was accompanied by a decrease in the threshold potential and a higher membrane resistance of WT-PCs in the presence of E-4031 and of *Kcnh7*-KO PCs (see legend to fig. S1). Together, we conclude that PCs lacking erg3 subunits are hyperexcitable.

To investigate whether synaptic potentials contribute to this increase in spontaneous activity, we performed experiments in the presence of synaptic blockers (DNQX, bicuculline, and AP5; see the Supplementary Materials), i.e., we blocked both inhibitory postsynaptic potentials and excitatory postsynaptic potentials. In on-cell recordings from WT-PCs, we found a significant increase in the frequency of spontaneous activity following application of synaptic blockers (fig. S2, B and D). After application of E-4031 in the presence of synaptic blockers, an additional increase in spontaneous firing occurred (fig. S2, C and D). These results indicate that under in vivo conditions, synaptic potentials have a dampening effect on the generation of spontaneous activity in PCs.

**Fig. 6.**
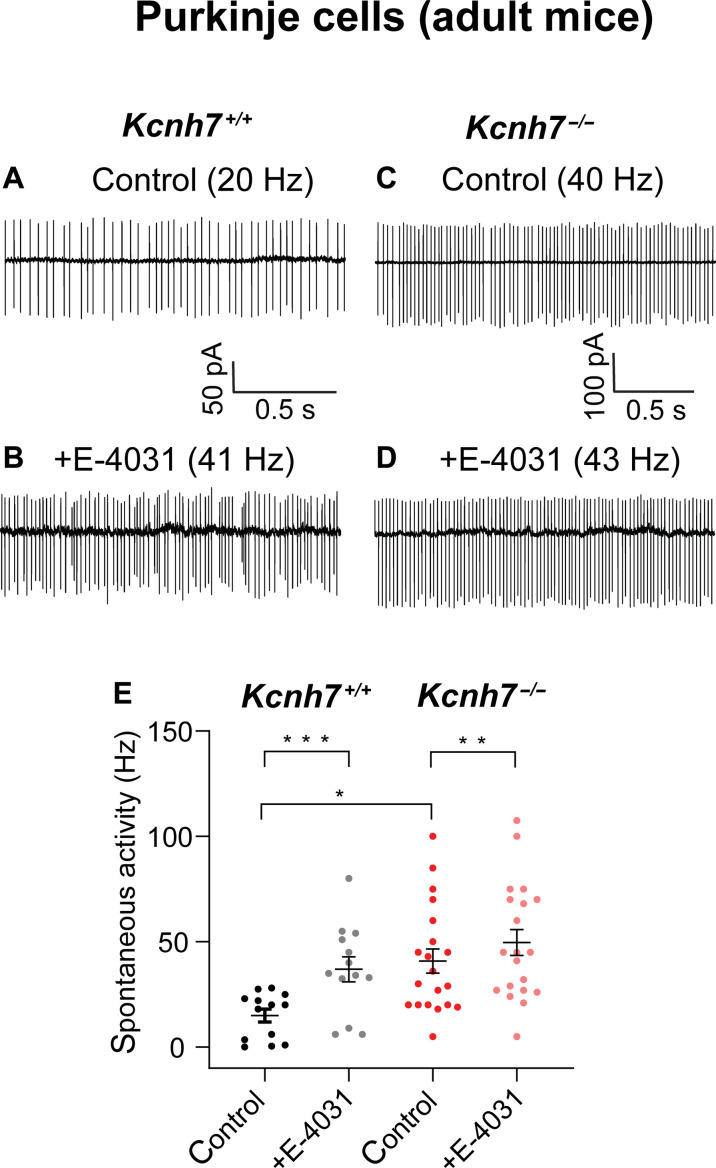
PCs from adult *Kcnh7*-KO mice (aged 4.5 to 6 months) show increased resting spontaneous activity. On-cell recordings of spontaneous activity from a PC of a WT mouse in the absence (**A**) and in the presence of E-4031 (**B**) and of a PC from a *Kcnh7*-KO mouse in the absence (**C**) and in the presence of E-4031 (**D**). Temperature, 36°C. (**E**) Graph plotting spontaneous activity recorded from PCs of WT mice (*n* = 13; data from three mice) and *Kcnh7*-KO mice (*n* = 20; data from three mice). Wilcoxon test used to calculate statistical significance. Mann-Whitney test used to calculate the significance between WT control and *Kcnh7*-KO control. *P < 0.05, **P < 0.01, ***P < 0.001.

Since the behavior experiments were performed with mice at the age of 4.5 to 6 months, we investigated spontaneous activity with on-cell recordings from PCs of mice at this age. On-cell recordings have the advantage, that changes in the content of the cytoplasm are avoided. In PCs from WT animals, we found a strong increase in action potential firing in the presence of E-4031 (WT control: 15.0 ± 3.0 Hz; +E-4031: 37.0 ± 5.9 Hz; *n* = 13; *P* < 0.001; Fig. 6, A, B, and E). Similar to PCs from adolescent mice, PCs from adult erg3-KO mice exhibited an increased resting frequency of action potential firing, with values significantly higher than the resting activity of WT mice. Notably, upon application of E-4031, there was a further increase in the frequency of action potential firing (*Kcnh7*-KO control: 40.9 ± 5.7 Hz; +E-4031: 49.6 ± 6.2 Hz, *n* = 20, 3 animals, *P* = 0.003; Fig. 6, C to E). Such an increase was not observed in adolescent animals (Fig. 5D) and is likely due to an E-4031–mediated blockage of erg1 channels that are still present in PCs of *Kcnh7*-KO mice. Together, we conclude that in WT-PCs, erg membrane currents are mediated by heteromeric erg1/erg3-containing K^+^ channels with a large contribution of erg3-type channels that contribute to the maintenance of the resting potential. A combination of erg3 and erg1 channel subtypes seems to regulate PC excitability, i.e., the frequency of their spontaneous action potential firing and repetitive activity.

### CA1 neurons from *Kcnh7*-KO mice are not hyperexcitable

**Fig. 7.**
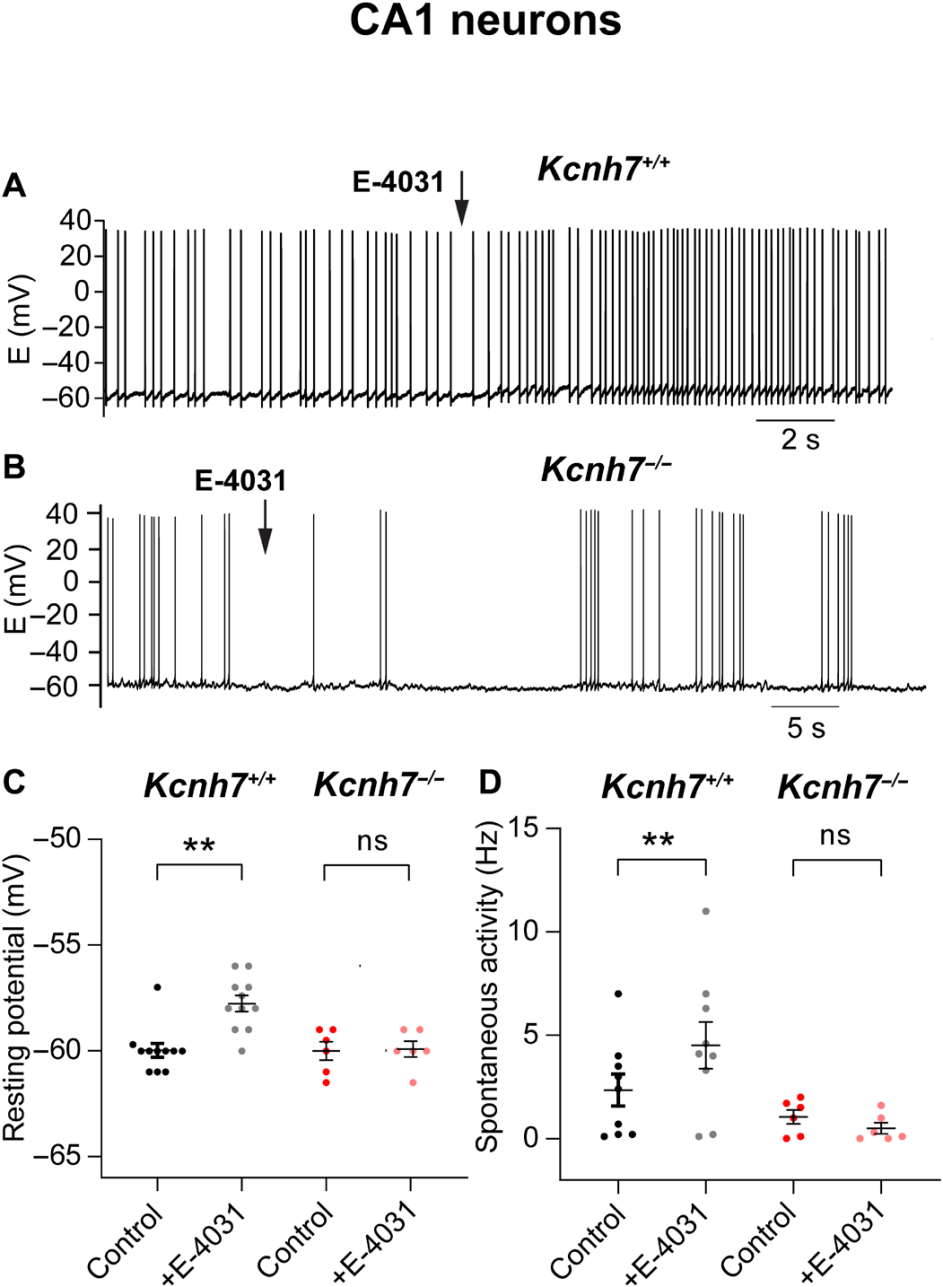
E-4031 increases spontaneous activity in WT CA1 neurons but not in CA1 neurons from *Kcnh7*-KO mice. (**A**) Current clamp recording of a CA1 neuron of a WT mouse (age, 2 weeks). Application of E-4031 induced a depolarization and an increase in spontaneous activity. (**B**) No change in spontaneous activity recorded from a CA1 neuron of a *Kcnh7*-KO mouse (age, 7 weeks) by application of E-4031. Temperature, 36°C. Graphs plotting the resting potential (**C**) and spontaneous activity (**D**) in CA1 neurons of WT mice in the absence (CA1-WT; resting potential, *n* = 11, data from four mice; spontaneous activity (*n* = 9; data from four mice) and presence of E-4031, as well as in CA1 neurons of *Kcnh7*-KO mice in the absence (CA1-KO; resting potential: *n* = 6, data from four mice; spontaneous activity: *n* = 6, data from four mice) and presence of E-4031. Calculation of statistical significance: resting potential (Wilcoxon test); spontaneous activity (paired *t* test). ns, not significant; **P < 0.01.

In situ hybridization data had shown that hippocampal CA1 neurons express mRNA transcripts of all three erg subunit genes (*4*, *7*). However, as reported above, we detected only erg3 channel–mediated K^+^ currents with no contribution of other erg subunits. We then measured the resting membrane potential and spontaneous activity in CA1 neurons (Fig. 7). Similar to WT-PCs, the resting potential of WT-CA1 neurons was close to −60 mV (*28*, *29*). Upon application of E-4031 (10 μM), the neurons depolarized by about 2 mV (controls: −60.0 ± 0.3 mV, +E-4031: −57.8 ± 0.4 mV, *n* = 11; *P* = 0.003; Fig. 7, A and C, black-gray) and generated or increased existing spontaneous irregular firing rates of action potentials at low frequency (controls: 2.3 ± 0.8 Hz, +E-4031: 4.5 ± 1.1 Hz; *n* = 9; *P* = 0.003; Fig. 7, A and D, black gray). This frequency of spontaneous firing was within the range reported for CA1 neurons (*28*). These results indicated that there are qualitative similarities to WT-PCs. In contrast, the resting potential of CA1 neurons from *Kcnh7*-KO mice was not influenced by the depletion of erg3 subunits and remained unchanged in the presence of E-4031 (10 μM) (controls: −60.0 ± 0.4 mV, +E-4031: −60.0 ± 0.4 mV, *n* = 6; Fig. 7, B and C, red-pink). Notably, and in contrast to PCs, we did not observe any increase in the resting spontaneous activity of CA1-KO neurons (controls: 1.1 ± 0.3 Hz; +E-4031: 0.5 ± 0.3 Hz, *n* = 6; Fig. 7, B and D, red-pink). These results obtained by whole-cell recordings were confirmed by on-cell measurements. In these on-cell recordings, spontaneous activity of WT-CA1 neurons increased significantly after erg channel blockage with E-4031, whereas E-4031 application had no effect on the low spontaneous activity of CA1 neurons from *Kcnh7*-KO mice (WT control: 2.3 ± 1.1 Hz; +E-4031: 7.6 ± 2.0 Hz, *n* = 14, data from five mice, age: postnatal day 10 (P10) to P17; *Kcnh7*-KO: 3.9 ± 1.5 Hz; +E-4031: 3.4 ± 1.1 Hz, *n* = 6, data from two mice, age: P10 to P17). This increase in spontaneous firing activity of WT-CA1 neurons in the presence of E-4031 was also found in the recording of repetitive activity in WT-CA1 neurons, whereas no influence of E-4031 was detected in CA1 neurons of *Kcnh7*-KO mice (fig. S3D).

Blocking synaptic potentials in WT-CA1 neurons by applying synaptic blockers slightly depolarized the resting potential (fig. S4D) and increased the frequency of spontaneous activity. However, both changes were not significant (fig. S4, D and E). Repetitive activity was also not altered by synaptic blockers in WT-CA1 neurons (fig. S4F) and CA1 neurons from erg3-KO mice (fig. S4G). In contrast, we observed an increase in repetitive firing in the presence of E-4031 and synaptic blockers in WT-CA1 neurons (fig. S4F).

The unaffected excitability of CA1 neurons from erg3-KO mice could be due to a compensatory increase of other subthreshold K^+^ currents, e.g., the M current or Kv1-mediated currents (*30*). We investigated whether the M current might be changed. The M current is mediated by KCNQ2/3 channels (*31*) and contributes to the maintenance of the resting potential (*28*). We compared the magnitude of the depolarization after application of 10 μM XE991 in CA1 neurons from WT and *Kcnh7*-KO mice. The depolarization in CA1 neurons from *Kcnh7*-KO mice was slightly larger than in WT-CA1 neurons (table S2). However, this difference was not statistically significant. Other types of K^+^ channels could mediate larger K^+^ currents in compensation for the lack of erg3 current (*30*). In support of the absence of subthreshold K^+^ channels in the axon initial segment of PCs (*32*), we found no depolarizing response to XE991 application in PCs (table S2). In conclusion, we show that CA1 neurons contain functional erg K^+^ channels formed by erg3 subunits only.

### Genetic depletion of erg3 K^+^ channel subunits reduces locomotor activity

Voltage-gated K^+^ channels are essential for neuronal excitability and behavior (*33*). Among them, erg K^+^ channels are still incompletely understood, and it is unknown whether and how they contribute to the regulation of behavior. We therefore studied adult WT mice and KO littermates lacking erg3 subunits using a broad spectrum of behavioral paradigms, including motor behavior, cognitive performance, and/or social interaction.

**Fig. 8.**
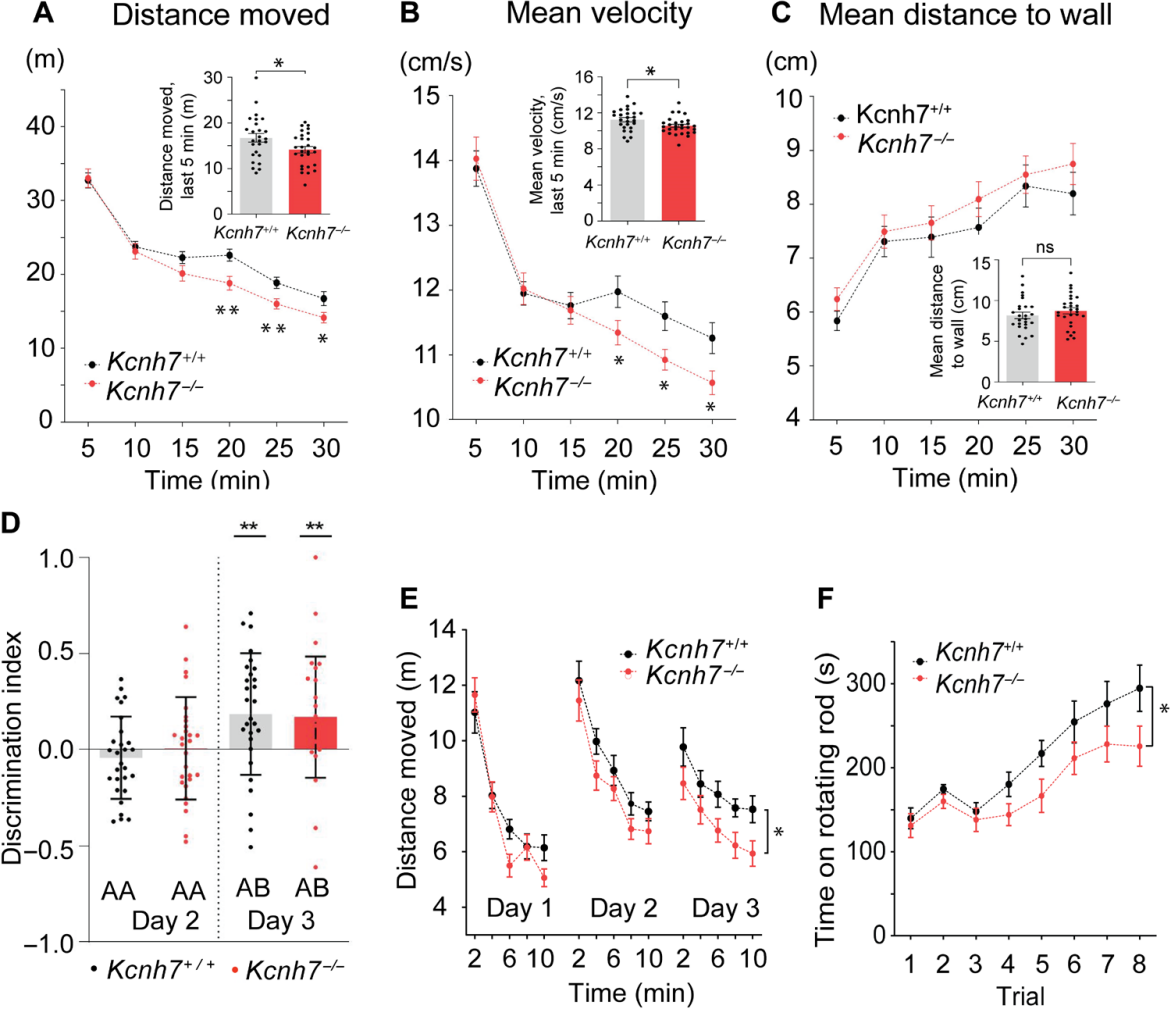
*Kcnh7*-KO mice show reduced locomotor activity. (**A** to **C**) Open field test. Reduced distance moved (A) and reduced mean velocity (B) in the second half of observation time. Unchanged mean distance to wall (C). (**D** and **E**) Novel object recognition test. (D) Measurement of time spent with two identical objects (AA) or one familiar and one new object (AB) on two successive days (day 2 and day 3). Day 1, no objects were presented. Pooled data of female and male mice. Calculation of discrimination index s. Material and Methods. (E) TDM of WT (black dots) and *Kcnh7*-KO mice (red dots) plotted against time (10 min divided in 2-min bins) on day 1, day 2, and day 3. *Kcnh7*-KO mice moved less on day 1 and day 2 (tendency) and significantly less on day 3. Statistical significance calculated by two-way repeated measures analysis of variance (ANOVA). (**F**) Rotarod test. Graph plotting time on the rotating wheel (s) against eight trials (1 to 8). Within the eight trials, time spent on the rod was measured under the following conditions: Day 1, two habituating runs with low constant speed (4 rpm) of the rods (1 and 2), and three trials with accelerating speed (4 to 40 rpm) of the rotating rods (3 to 5) were performed [inter-trial interval (ITI) 50 to 60 min]. Day 2, three trials (6 to 8) with accelerating rods (4 to 40 rpm) were performed (ITI, 15 min). Plots of pooled results of female and male WT mice (black dots) and *Kcnh7*-KO mice (red dots). Significant difference of the two curves as calculated by the two-way repeated measures ANOVA. ns, not significant; *P < 0.05, **P < 0.01.

In the first experiment, we investigated exploration behavior and overall activity levels using the open field test. We found that *Kcnh7*-KO mice moved similar distances to the WT controls, in particular, during the first half of the test period, but moved significantly shorter distances during the second half of the observation time (Fig. 8A). The mean velocity of KO animals was also significantly decreased over time (Fig. 8B), although the mean distance to the walls remained the same (Fig. 8C). In a second set of experiments, we tested anxiety (elevated plus maze) and social recognition and found that all genotypes performed equally well. Likewise, in the novel object recognition test, both WT and *Kcnh7*-KOs spent similar amounts of time investigating two identical objects on experimental day 2 (Fig. 8D, left) and spent significantly more time in exploring the new object on day 3 (Fig. 8D, right). This suggests, that K^+^ channels containing erg3 subunits do not contribute to the regulation of recognition memory. Nevertheless, *Kcnh7*-KO mice moved significantly less during day 3 of the novel recognition test (Fig. 8E), similarly as observed in the open field arena (compare with Fig. 8A), suggesting a general decrease in activity or a decrease in locomotor activity.

**Fig. 9.**
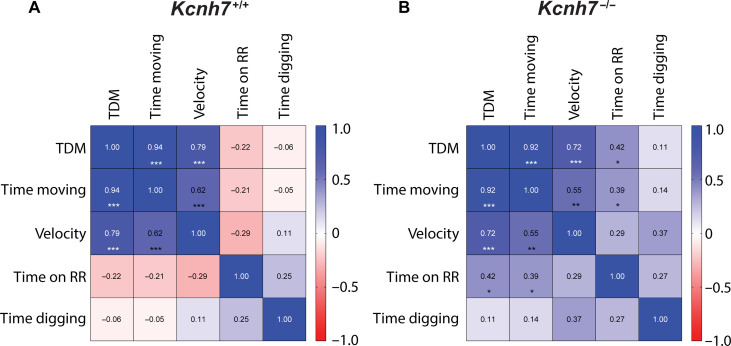
Multicorrelation matrix suggests a general reduction of locomotor activity. Multicorrelation matrix of WT (**A**) and *Kcnh7*-KO (**B**) mice. Mean of TDM (all tests), mean % of time moving (time moving, all tests), and mean of velocity calculated from all motor tests (velocity; tests included, open field test, elevated plus maze, novel object recognition test, and social interaction test), as well as the motor parameters of the rotarod test (time on RR) and time digging (time digging). Numbers in the rectangular fields are the Pearson coefficients and the level of significance (*, **, ***). On the right side of each matrix, significance level from blue (1.0) to red (−1.0).

These data prompted us to investigate motor behavior using the rotarod test, which can provide information on motor balance, coordination, grip strength, and endurance. The performance of KO mice lacking erg3 subunits on the accelerating rod [(4 to 40 rounds per minute (rpm)] was found to be significantly lower compared to WT control animals (Fig. 8F). Initially, during the first two trials, WT and *Kcnh7*-KOs performed equally well at low constant speed. In contrast, with increasing speed and shorter time intervals between trials, erg3 KOs remained on the wheel significantly shorter times (*P* = 0.021), a finding that correlates with the aberrant neuronal excitability in PCs of *Kcnh7*-KO mice (Figs. 5 and 6). A general tendency to reduced motor activity following erg3 subunit depletion was confirmed in a multicorrelation matrix comparing the results of multiple behavioral tests across genotypes (Fig. 9). As revealed by Pearson’s correlation coefficients, the matrix of KO mice (Fig. 9B) revealed a statistically significant positive correlation between the times spent on the rotarod and the total distance moved (TDM, 0.42) or the time spent moving (0.39), whereas no such correlation was detected with WT mice (TDM −0.22; time moving −0.21; Fig. 9A). These data suggest that erg3 KO mice are characterized by a general reduction in motor performance and/or endurance.

**Fig. 10.**
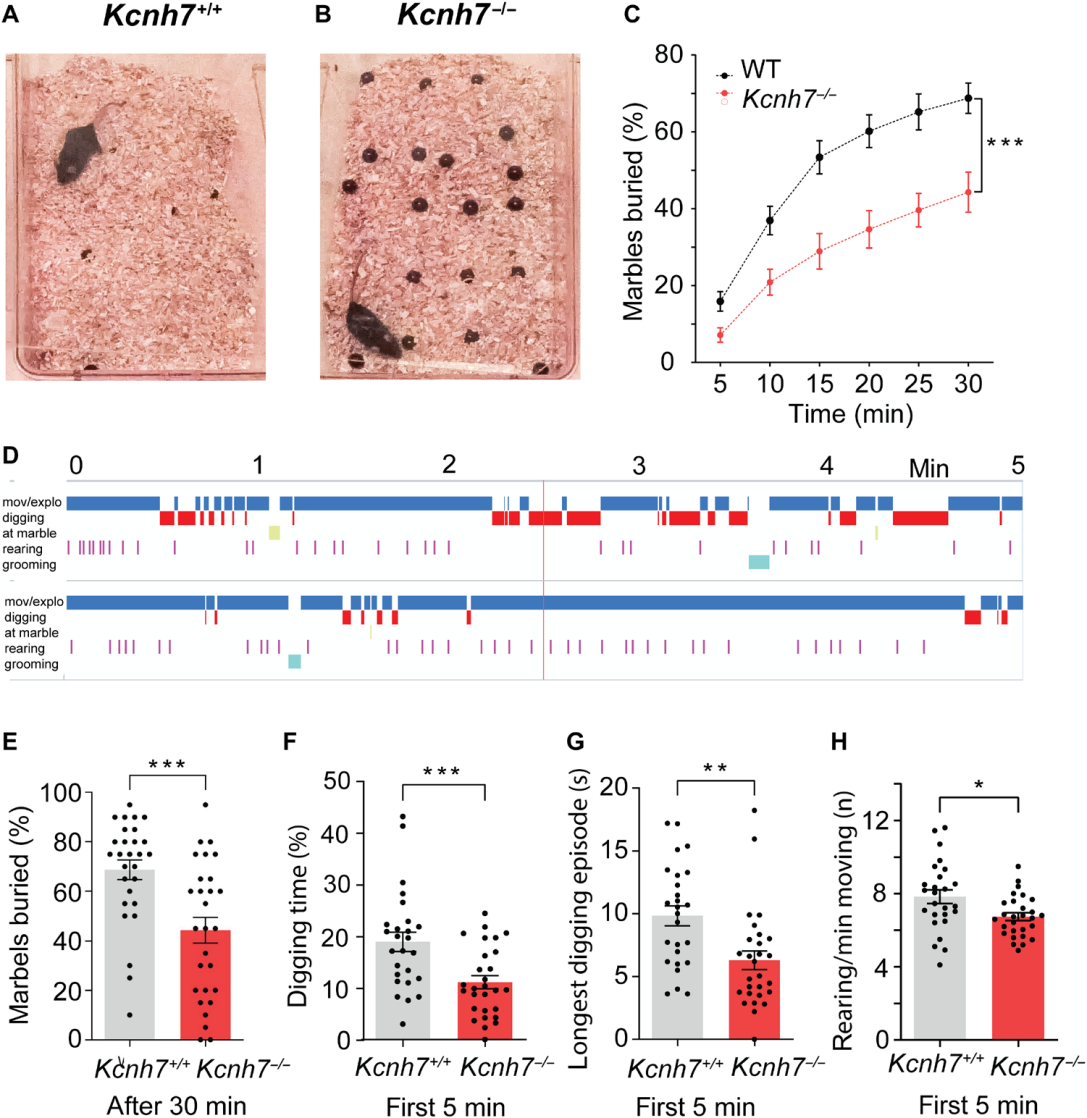
Strongly reduced locomotor activity (marble burying test) in *Kcnh7*-KO mice. Representative photographs of cages containing a WT mouse (**A**) and *Kcnh7*-KO mouse (**B**) at the end of the 30-min test time. Cages contained bedding of about 5-cm height and 20 black marbles regularly distributed on the bedding before inserting the mice into the cages. (**C**) Graph plotting percentage of buried marbles (in %) in bins of 5 min in WT mice (black dots) and *Kcnh7*-KO mice (red dots). Symbols connected by straight lines. The two datasets are significantly different, as calculated by the two-way repeated measures ANOVA. (**D**) Ethograms of a WT mouse (top) and *Kcnh7*-KO mouse (bottom). Evaluation of marbles buried after 30 min (**E**), digging time (%) during first 5 min (**F**), longest digging episode during first 5 min (**G**), and number of rearing/min during the first 5 min (**H**) of WT and *Kcnh7*-KO mice. *P < 0.05, **P < 0.01, ***P < 0.001.

To further investigate this possibility, we tested the digging behavior of WT and *Kcnh7*-KO mice by applying the marble burying test, an assay used to test motor function and repetitive behavior (*34*, *35*). WT mice expressing erg3 subunits buried about 70% of all marbles within 30 min (Fig. 10, C and E). *Kcnh7*-KO mice showed significantly less digging across the test period and consequently buried far fewer marbles over time (Fig. 10, A to D). WT control animals showed a frequent and vigorous digging behavior that was barely detectable in *Kcnh7*-KO animals. They spent more time digging compared to *Kcnh7*-KO mice (Fig. 10, F and G), and even the number of rearing events was significantly lower in *Kcnh7*-KO mice (Fig. 10H). Since digging is assumed to be an endogenous repetitive motor activity, the number of marbles buried may be an indicator of vigor (*35*). In summary, we conclude that depletion of the erg3 subunits leads to deficits in locomotor function, endurance, and vigor, reminiscent of a depressive-like behavior.

## DISCUSSION

In this study, we have generated two new mouse lines characterized by either a PC-specific KO of the erg1 gene or a global KO of the erg3 gene, both encoding essential subunits in the formation of erg-type K^+^ channels. Using electrophysiology, immunochemistry, and mouse behavioral analysis, we showed that erg K^+^ currents of cerebellar PCs are composed of two components mediated by erg1 and erg3 subunits. In contrast, CA1 neurons of the hippocampus are characterized by pure erg3 K^+^ currents. At the single-neuron level, we found that erg3 subunits mediate subthreshold currents that activate near the resting membrane potential and modulate spontaneous and repetitive activity in WT-PCs and WT-CA1 neurons. Genetic depletion of erg3 subunits was associated with hyperexcitability in PCs but not in CA1 neurons. These two types of neuronal responses to an erg3 subunit depletion, hyperexcitability, and normal excitability may represent two extremes of a broad spectrum of excitability changes. At the behavioral level, the absence of erg3-mediated K^+^ currents resulted in a general attenuation of locomotor activity. *Kcnh7*-KO mice lacking erg3 subunits showed significantly reduced performance on an accelerating rotating wheel and a continuously weaker motor behavior in the open field. They also showed significantly reduced activity in repetitive movement such as digging. In contrast, *Kcnh7*-KO mice showed normal performance in learning and memory as well as in recognizing social or novel objects.

### The erg3 K^+^ current is a subthreshold current

Our data confirm our previous findings that in cerebellar WT-PCs, erg currents are likely mediated by heteromeric erg1/erg3 K^+^ channels (*15*). We now show that in PCs, erg3-mediated current amplitudes are larger than erg1-mediated current amplitudes, suggesting that erg3 K^+^ channel subunits predominate in the composition of heteromeric erg1/erg3 channels (*3*). Homomeric erg3 channels are weak inward rectifiers, which mediate a small sustained outward current. Therefore, they are likely to contribute to its maintenance. These observations may explain why erg channel blockade by E-4031 depolarizes PCs and initiates or enhances spontaneous firing of action potentials. In PCs, erg channel-mediated currents are the dominant subthreshold K^+^ currents; for instance, their axon initial segments do not contain any other subthreshold K^+^ channels (*32*, *36*–*38*). Accordingly, PCs lacking erg3 K^+^ channel subunits show persistent hyperexcitability, and the remaining homomeric erg1 K^+^ channels in these neurons cannot reduce their hyperexcitability since they are either activated at more positive membrane potentials and/or their current amplitudes are too small to be effective. In hippocampal WT- CA1 neurons, erg K^+^ currents are likely mediated by homomeric erg3 K^+^ channels as no other erg current is detectable. These data support recent findings, suggesting that erg3 K^+^ channels are dominant in hippocampal CA1 neurons (*17*). Acute blockage of erg3-containing K^+^ channels by E-4031 depolarized CA1 neurons and increased their spontaneous activity as well as their repetitive activity, similar to that observed in PCs. The fact that the lack of erg3 subunits has no functional consequence for the excitability of CA1 neurons was confirmed by on-cell recordings. This suggests that in CA1 neurons, other types of K^+^ channels may be up-regulated during development and may compensate for the loss of erg3 in *Kcnh7*-KO mice. One candidate for a functional adaptation in CA1 neurons is the M current (*28*). However, we could not find an increased depolarization in CA1 neurons of erg3 KO mice as compared to WT CA1 neurons (table S2). In inferior olivary neurons, erg3 subunit–containing channels were found to exhibit an inductor-like property that mediates resonance and membrane oscillations near the resting potential (*21*). This ability enables them to support the generation of subthreshold oscillations and spontaneous activity (*39*, *40*). Erg1 K^+^ channels do not have this property (*21*).

### Erg1-containing K^+^ channels mediate a suprathreshold current

Compared to erg3 K^+^ channels, erg1 K^+^ channels are activated at more positive membrane potentials and convey tiny outward currents due to their strong inward rectifier properties (Fig. 4) (*10*). Consequently, in PCs of young *Kcnh7*-KO mice lacking erg3 subunit containing K^+^ channels, the high frequency of resting spontaneous activity was not further increased after E-4031–mediated blockage of the remaining erg1 K^+^ channels (Fig. 5). However, in adult *Kcnh7*-KO mice, E-4031 blockage of erg1-containing K^+^ channels increased the frequency of spontaneous firing (Fig. 6), possibly due to a compensatory increase in erg1 channel density. Up-regulation of erg1 subunit gene expression occurs in the vomero-nasal organ following odor stimulation of mice by pheromones (*18*). An important neuronal function of erg1 K^+^ channels is the generation of accommodation during repetitive activity. Because of their slow activation kinetics, interspike intervals become longer and longer until no action potential is generated anymore (*9*, *41*). In contrast, since erg3-mediated K^+^ currents have faster gating kinetics, they just increase the interspike intervals during repetitive activity without inducing accommodation (*15*). In dopaminergic neurons, activation of erg1-type K^+^ channels can terminate burst activity, as suggested by the observation that E-4031 converts burst firing into spontaneous irregular tonic firing (*13*). Furthermore, activation of the erg1 current in bursting dopamine neurons shortens the action potential plateau duration, similarly as the HERG current shortens the cardiac action potential (*19*).

### Genetic depletion of erg3-type K^+^ channels attenuates locomotor activity

In situ hybridization and immunocytochemistry have shown that erg1 and erg3 K^+^ channels are widely expressed throughout the rodent brain (*4*–*7*). It is therefore unexpected that the global lack of erg3 subunits leads to a reduction in locomotor activity, suggesting that erg1 and possibly also erg2 channel subunits do not functionally compensate for the absence of erg3 subunits. *Kcnh7*-KO mice lacking erg3 subunits were found to have weaker locomotor activity in various tests. For example, they spent less time on a rotating wheel and showed a remarkably strong reduction in digging behavior (Fig. 10). Digging consists of repetitive leg movements that are regulated by neuronal circuits involving the cerebellum, basal ganglia, brainstem, and spinal cord (*35*, *42*). This test has previously been used to investigate the efficacy of anxiolytic drugs such as benzodiazepines (*35*). *Kcnh7*-KO mice lacking erg3 subunits appeared as if they had been treated with a psychoactive drug such as the serotonin reuptake inhibitor fluoxetine or the antipsychotic drug haloperidol; both drugs are known to block erg channels (*16*, *43*). Marbles have also been discussed as aversive objects for mice that induce anxiety suggesting that digging might represent an obsessive-compulsive disorder–like symptom (*35*, *44*). In the present study, we interpret the decreased digging activity together with the decreased activity in the open field and rotarod tests as the consequence of a general depressive-like behavior in *Kcnh7*-KO. This view is supported by the positive correlations between different motor tasks in the multicorrelation matrix in *Kcnh7*-KO (Fig. 9). Traditionally, PCs transmit their inhibitory activity to deep cerebellar and vestibular nuclei, thereby controlling body balance, motor coordination, and motor learning (*42*, *45*). In addition, there are connections from the cerebellum to the premotor and motor cortices and cingulate gyrus (*46*). Recently, it has been reported that PC hyperexcitability induced by acute local inflammation of the rat cerebellar cortex is associated with multiple depressive-like behaviors (*47*), which are very similar to those we observed in *Kcnh7*-KO mice. In contrast, cerebellar neurodegeneration induces decreased excitability of PCs associated with motor symptoms like ataxia (*46*). Since we have studied mice with a global KO of erg3 subunits, their behavioral changes are very likely the result of activity changes in a large number of different types of neurons, in addition to PCs and CA1 neurons.

CA1 neurons of the hippocampus are part of the limbic system and receive input from the entorhinal cortex, the dentate gyrus, CA2 and CA3 neurons. The hippocampus is responsible for spatial representation of the environment in the brain as well as for learning and episodic memory (*48*). Erg3-mediated K^+^ channel currents attenuate the excitability of CA1 neurons in WT mice but not in *Kcnh7*-KO mice. It is therefore plausible that the absence of erg3-mediated K^+^ channels is compensated either by up-regulation of erg1 and/or erg2 or, more likely, by other non-erg–type subthreshold K^+^ channels (*28*, *49*). Unfortunately, available antibodies are currently unable to discriminate between individual erg-subunits, so alternative analyses are required to gain insights into the molecular composition of *Kcnh7*-KOs. Omics analyses and/or magnetic resonance tomography (*50*) could help to study gene expression patterns and network activity patterns in mutant mice.

A primate-specific isoform of erg1a (*KCNH2-3.1*) has been detected in the brains of schizophrenic patients (*51*), and erg3-containing K^+^ channels have been discussed in the context of bipolar mood disorder and epilepsy. In a cohort of patients from an Amish community in Pennsylvania, 14 of 26 patients diagnosed with bipolar mood disorder carried an Arg394His point mutation in the human *KCNH7* gene, which encodes erg3 (*52*). Electrophysiology after heterologous expression of mutant erg3(Arg394His) revealed smaller current amplitudes together with an activation curve shifted to more positive membrane potentials (*52*). These biophysical changes resulted in a reduction up to a functional loss of erg3 subunit–mediated K^+^ channel currents. Experiments using down-regulation of erg3 gene expression by using small interfering RNA have demonstrated enhancing epileptic seizure susceptibility in mice (*17*). The same study reported that erg3 expression is reduced in epileptic foci of patients with temporal lobe epilepsy. However, in contrast to mice lacking hippocampal KCNQ2/3-containing K^+^ channels (*49*), *Kcnh7-*KO mice lacking erg3 subunits in the present study did not develop epileptic seizures. We therefore conclude that compensatory mechanisms step in to maintain functional erg-mediated K^+^ channel currents, underlining the importance of this particular K^+^ channel subfamily in the brain.

In summary, we found that the erg current of PCs contains erg3 and erg1 currents, whereas that of CA1 neurons is a pure erg3 current. We show that in both neuron types, the erg3 current is activated near the resting membrane potential, thereby dampening spontaneous firing of action potentials. The excitability of neurons in mice with a global KO of erg3 subunits depends on the neuron type, in PCs, excitability is increased, and that of hippocampal CA1 neurons is normal. On the behavioral level, neuronal hyperexcitability of PCs and presumably that of other neurons induce a general weakness and attenuation of locomotor activity that appears as depressive-like behavior.

## MATERIALS AND METHODS

### Generation of conditional *Kcnh2* KO mice lacking erg1 subunits in PCs

*Kcnh2* encodes erg1a and four isoforms (erg1b, erg1-3.1, erg1a_uso_, and erg1b_uso_), all of which comprise exons 6 to 9. We used the Cre/Lox technology to generate a conditional KO of *Kcnh2* following the procedure previously described (*20*). We obtained *Kcnh2*^tm1a(EUCOMM)Wtsi^ (reporter-tagged insertion with conditional potential) embryonic stem (ES) cells from the European Mouse Mutant Cell Repository containing floxed exons 6 to 9 of *Kcnh2* (*53*). After injection of ES cells into blastocysts, they were implanted into pseudopregnant mice yielding offspring containing a floxed *Kcnh2* (B6-*Kcnh2^flox^*). To generate mice with a PC-specific *Kcnh2* KO, we crossed the floxed mice with bacterial artificial chromosome transgenic mice carrying the cre recombinase gene under control of the PC-specific *L7/pcp2*-promoter (B6.Cg-Tg(Pcp2-cre)3555Jdhu/J mice; stock no. 010536, The Jackson Laboratory) (*25*, *54*, *55*).

### Generation of global *Kcnh7*-KO mice via CRISPR-Cas9

To generate the *Kcnh7* KO mouse line, two single-guide RNAs (sgRNA) were chosen after submitting the targeting region around exon 5 of *Kcnh7 t*o the CRISPOR design tool (http://crispor.tefor.net). Templates for transcription with the targeting sequences (TCCTGAAGTAACCCCCAACA) and (CATAGTCTTCTCTCTAATAG) were generated by a fill-in reaction with Klenow DNA polymerase (Thermo Fisher Scientific). Transcription was performed using the HiScribe T7 High Yield RNA Synthesis Kit (#E2040S, New England Biolabs), with subsequent purification of the transcript with the MEGAClear Transcription Clean-Up Kit (#AM1908, Thermo Fisher Scientific), both according to the manufacturer’s instructions.

The sgRNAs and Cas9 protein (Alt-R S.p. Cas9 Nuclease V3, #1,081,058, Integrated DNA Technologies, Leuven, Belgium) (500 ng/μl) in Gibco Opti-MEM (Thermo Fisher Scientific) were used for electroporation into one-cell stage embryos derived from superovulated C57BL/6JUke mice using the NEPA 21 electroporator [Nepa Gene, Ichikawa-City, Japan; for settings, see (*56*)] and implanted into foster mice. The resulting offspring was analyzed by polymerase chain reaction (PCR) using primer A: GTA GAG ACT CCG TGG ATC ATT TCA TAT AGG TA and primer B: CCA AGT ATG ATG AAT AGC TCA GTA ATT ATT TCA GAG CA. Deletions were verified by sequence analysis and an additional PCR with primer A and WT-specific primer C (GTT TGA ATC TGA TGT GGA TCC CAG C). One of four founder mice was chosen to establish the mouse line B6-KCNH7^em2Hhtg^ (*Kcnh7*-KO). Exon 5 deletion was detected by PCR in heterozygous (+/−) and homozygous (−/−) genotypes (Fig. 1B). To confirm the absence of erg3 protein, we first applied immunoprecipitation with an erg3-specific antibody using brain lysate. While erg3 was efficiently precipitated in WT (+/+) tissue, the protein was undetectable in brain lysate derived from homozygous (−/−) littermates (Fig. 1C). Comparison of Nissl stainings of a WT mouse (left) and a Kcnh7^−/−^ mouse (right) revealed no obvious anatomical defects (Fig. 1D).

### Genotyping of global *Kcnh7*

For PCR genotyping of offspring mice, the WT allele was detected by the forward and reverse primers (A + C) resulting in a 566–base pair (bp) fragment, as well as primer A + B for a 977-bp fragment, which was not obtained under the used conditions. The KO allele was detected by primer A + B resulting in a 328-bp fragment. DNA fragments were separated on 1.2% agarose gels and imaged using an ultraviolet transillumination/digital camera system (INTAS Science Imaging Instruments). Absence of erg3 channel protein in *Kcnh7^−/−^* mice was confirmed by Western blot using pre-adsorbed anti-*Kcnh7* antibody and extracts of hippocampus and cerebellum of young and adult mice (Fig. 1C).

### Genotyping of conditional *Kcnh2*

To distinguish the successful removal of the sequence in-between the *Frt* sites of *Kcnh2*^tm1a(EUCOMM)Wtsi^ mice, we obtained a 601-bp DNA fragment detected with primer D + E, while the targeted allele was detected with primers E + F resulting in a 1016-bp DNA fragment. WT alleles were obtained by primers D + E, resulting in a DNA fragment of 433 bp. After applying our breeding strategy, we obtained animals with an *L7*-driven deletion of the conditional (floxed) *Kcnh2*^flox^ specifically in the cerebellum. We detected Cre expression by PCR with primer G + H resulting in a DNA fragment of 650 bp.

### Western blot

For the detection of erg1 and erg3 channel proteins in cerebellar crude extracts (Fig. 1C), single cerebella were lysed in ice-cold lysis buffer [20 mM Hepes, 100 mM potassium actetate, 40 mM KCl, 5 mM EGTA, 5 mM MgCl_2_, 1% Triton X-100, and protease inhibitors (cOmplete Protease Inhibitor Cocktail; #04693116001, Roche)] using 12 strokes with Potter S Homogenizer (Sartorius AG). The homogenate was centrifuged at 1000*g* for 10 min at 4°C, and the supernatant was mixed with 20% (v/v) loading dye [500 mM dithiothreitol. 0.05% bromophenol blue, 50% glycerol, 10% SDS, and 250 mM tris-Cl (pH 6.8) and denatured (5 min, 97°C]. Antibodies used are as follows: anti-KCNH7 rb (alomone #APC112, RRID: AB_2039937), anti-KCNH7 rb (Thermo Fisher Scientific, #PA5-68276, RRID: AB_2691815), anti-KCNH7 rb (ProteinTech, #13622, RRID: AB_10638620), and anti HERG1 rb (Merck Millipore, # AB5930, RRID: AB_92144).

### Nissl staining

Twenty-five–micrometer sagittal sections were sequentially rinsed in H_2_O, 70% ethanol, 95% ethanol, 100% ethanol, and xylene to delipidize the tissue. To rehydrate sections were sequentially rinsed in xylene, 100% ethanol, 95% ethanol, 70% ethanol, and H_2_O. Sections were dipped and gently agitated in 0,1% Cresylviolett in 0.25% acetic acid. Excess stain was rinsed off in H_2_O followed by a sequential dipping into 70% ethanol, 95% ethanol, 95% acidic ethanol, 100% ethanol, and xylene. Sections were then mounted with Entellan, and images were acquired with a stereomicroscope (Zeiss, Stemi 2000C).

### Immunohistochemistry

For immunohistochemistry, 12- to 16-week-old mice were euthanized and decapitated. The brains were fixed overnight at 4°C with 4% paraformaldehyde before 25-μm sections were cut. The brain sections were washed in phosphate-buffered saline, blocked, and permeabilized for 1 hour at room temperature in 100 mM phosphate buffer (PB) containing 10% normal donkey serum (NDS), 0.3% Triton X-100, and 150 mM NaCl. Immunostaining was performed overnight at 4°C with commercially available primary antibodies detecting Kv11.1 (1:400; Alomone Labs: #APC-016, RRID: AB_2039911), calbindin (1:300; Sigma-Aldrich: #C9848, RRID: AB_476894), and ankyrin G (1:300; Synaptic Systems: #386 004, RRID: AB_2725774) in 100 mM PB containing 2% NDS, 0.05% Triton X-100, and 150 mM NaCl. Sections were washed three times with PB and incubated with secondary antibodies [donkey anti-rabbit cy3 (Dianova: #711-166-152, RRID: AB_2313568), donkey anti-mouse Alexa-488 (Dianova: #715-546-150, RRID: AB_2340849), and donkey anti-guinea pig cy5 (Dianova: #706-175-148, RRID: AB_2340462)] in 100 mM PB containing 2% NDS, 0.05% Triton X-100, and 150 mM NaCl for 2 hours. Sections were washed three times with PB and mounted with Aqua Polymount (Polysciences Inc.). Images were aquired with an Olympus FV1000 confocal laser scanning microscope (60× objective; 1-μm Z-plane thickness).

### Acute slice preparation

Slices were prepared from brains of male or female C57BL/6 mice (WT and *Kcnh7*-KO mice; age: 6 to 12 days or 3 to 25 weeks old). In accordance with the institutional guidelines of the University Hospital of Hamburg-Eppendorf, University of Hamburg, the mice were anesthetized with isoflurane and decapitated. The brain was removed from the skull and transferred into ice-cold carbonated (95% O_2_ and 5% CO_2_) sucrose slicing solution. For the cerebellar slices, the cerebellum was separated from the cortex, and the lateral part of one hemisphere was cut in the parasagittal plane, glued with cyanoacrylate on the cutting plate of a microtome (Leica VT 1200S), and transferred into the cutting chamber with cold carbonated (95% O_2_, 5% CO_2_) sucrose slicing solution. For horizontal hippocampal slices, the cerebellum and the prefrontal cortex were cut off, and the brain was divided into its two hemispheres; these were flipped onto their midplanes, and the dorsal part of the cortex was cut and glued onto the holder. Slices of 250 μm of the cerebellum or the hippocampus were cut and subsequently incubated in standard artificial cerebrospinal fluid (ACSF) at 35°C for 1 hour and, thereafter, at room temperature. The recordings were made within 8 hours after incubation. Sucrose slicing solution contained (in mM): 240 sucrose, 10 glucose, 26 NaHCO_3_, 1.25 NaH_2_PO_4_, 2.5 KCl, 2 MgSO_4_, and 1 CaCl_2_, carbonated with 95% O_2_ and 5% CO_2._

### Heterologous expression of erg1a and erg3 in HEK cells

For stable transfection, cDNA of erg1a or erg3 channels was linearized with MeuI (Thermo Fisher Scientific) and transfected into HEK293 cells (RRID: CVCL_0045) with calcium phosphate. Stable expression was achieved by using geneticin (Sigma-Aldrich). For electrophysiological measurements, the cells were placed on poly-l-lysine–coated glass coverslips and used within 1 to 3 days.

### Electrophysiology

On-cell and whole-cell patch clamp recordings in acute slices of the cerebellum or hippocampus were performed with an EPC10 amplifier (HEKA Electronics, 72770 Reutlingen, Germany). Neurons in acute slices were visualized with a water-immersed 40× objective of a Zeiss Axioskop2 FS plus microscope and infrared differential interference contrast optics. Borosilicate glass electrodes (Hilgenberg, 34323 Malsfeld, Germany; Science Products, 65710 Hofheim, Germany) were pulled (Flaming/Brown micropipette puller, model P-97, Sutter Instrument, Science Products) and filled with intracellular solution containing (in mM): 123 K^+^-methanesulphonate, 9 NaCl, 9 Hepes, 1.8 MgCl_2_, 0.9 EGTA, 14 Na_2_-phosphocreatine, 4 MgATP, and 0.3 Na_3_GTP, pH adjusted to 7.3 with KOH. Electrodes filled with intracellular solution had a resistance of 4 to 6 MΩ. Liquid junction potentials were not corrected. Only recordings with an access resistance <30 megohm were evaluated. Slices were continuously perfused (2.5 ml/min) with carbonated ACSF. Standard ACSF contained (in mM): 125 NaCl_2_, 20 glucose, 26 NaHCO_3_, 1.25 NaH_2_PO_4_, 2.5 KCl, 1 MgCl_2_, and 2 CaCl_2_, carbonated continuously with 95% O_2_ and 5% CO_2_ to reach a pH of 7.4. Voltage clamp recordings were made in a modified ACSF with increased K concentration containing (in mM): 87.5 NaCl, 20 glucose, 26 NaHCO_3_, 1.25 NaH_2_PO_4_, 40 KCl, and 3 MgCl_2_ carbonated continuously with 95% O_2_ and 5% CO_2_. Voltage clamp recordings were performed in the presence of 100 μM picrotoxin (P1675, Sigma-Aldrich) or 20 μM bicuculline (66016-70-4, TOCRIS), 50 μM AP-5 (79055-68-8, Tocris), and 1 μM tetrodotoxin (1078, Tocris). Current clamp and on-cell recordings were made at 32° to 36°C and voltage clamp recordings at room temperature (22° to 24°C). Some of the current clamp and on-cell recordings were also made in the presence of substances blocking synaptic activity. These substances were identical to those listed above. In addition, we performed current clamp recordings by inhibiting the M current with 10 μM XE991 (122955-13-9, TOCRIS). Recordings were low-pass filtered at 2.9 kHz.

Whole-cell patch clamp measurements on HEK293 cells permanently expressing erg1a or erg3 channels were performed using borosilicate pipettes with resistances of 3.0 to 4.5 megohm after filling with the same intracellular solution as used in recordings from neurons in acute slices. Patchmaster software (HEKA, Lambrecht, Germany) in combination with an EPC-9 patch-clamp amplifier (HEKA) was used for data acquisition and pulse application. Recordings were low-pass filtered at 2.9 kHz. HEK293 cells with an access resistance <20 megohm were evaluated. Voltage clamp experiments were done at room temperature (21° to 23°C) in normal Ringer’s solution (140 mM NaCl, 5 mM KCl, 2 mM MgCl_2_, 1 mM CaCl_2_, 10 mM Hepes, and 5 mM glucose, and pH adjusted to 7.3 with NaOH) or in a modified Ringer’s solution with an increased K concentration (100 mM NaCl, 40 mM KCl, 2 mM MgCl_2_, 10 mM Hepes, and 5 mM glucose, and pH adjusted to 7.3 with NaOH). If not otherwise stated, all substances were purchased from Sigma-Aldrich. Recordings were analyzed with Fitmaster (HEKA), Igor Pro 6.03 (Wavemetrics), and Excel (Microsoft).

### Mouse behavior

To investigate behavior, we performed a spectrum of seven tests. All mice underwent the tests in the same sequence. First, we measured explorative behavior, anxiety, and habituation (open field test and elevated plus maze); thereafter, we tested explorative behavior combined with learning and memory (novel object and social recognition test), followed by testing of motor coordination and motor skills (rotarod test and pole test). Last, we measured digging and burying of marbles as one type of endogenous locomotor activity (marble burying test). Tests were performed with one cohort of WT mice and one cohort of *Kcnh7*-KO mice; each cohort consisted of 28 animals (14 female and 14 male mice). The age of mice was between 4.5 and 6 months. GPower 3.1 was used to determine the number of animals of each cohort.

At the beginning of experiments animals were between 8 and 25 weeks old, and the mean age of these mice was 15.4 ± 0.4 weeks (means ± SEM). The mice were bred in a heterozygous manner in the animal facility of the Center for Molecular Neurobiology Hamburg at the University Medical Center Hamburg-Eppendorf. The animals were housed in groups of littermates (three to five individuals per cage) in an acclimatized animal vivarium (21° ± 1°C, relative humidity 55 ± 5%) under a reversed light/dark cycle 12:12 hours. The mice were tested during dark hours, and they had access to food and water ad libitum. The genotype of mice was blinded to the investigators during the performance of the experiments and their evaluation.

#### Open field test

The open field apparatus consisted of four arenas (50 cm by 50 cm by 50 cm) made of white polyvinyl foam material. Four lamps were installed providing even lighting (50 lux) in each arena, and a video camera was mounted directly above the apparatus. The videos were transmitted to a personal computer running Ethovision tracking software (Version XT8.5, Noldus Technology, the Netherlands). Up to four mice were tested at the same time, counterbalanced across genotypes but blind to the experimenter. The mice were introduced to one corner of the arena and were allowed to explore the arena undisturbed for 30 min. TDM, velocity, and mean distance to the wall were analyzed in 5-min consecutive time bins. The open field test investigates the general locomotor activity of mice as well as the balance between their tendency to stay near the wall of the arena where they feel safe and their curiosity to explore the center of the arena.

#### Elevated plus maze

The elevated plus maze was made of polyvinyl foam material and contained a removable white plastic floor. It was elevated 80 cm above the floor and illuminated with diffuse dim light (25 lux). The maze consisted of four equally spaced arms (length, 30 cm; width, 5 cm) radiating from a central square (5 cm by 5 cm). One pair of opposing arms was enclosed with opaque walls, whereas the remaining two arms were exposed with a 2-mm high perimeter border along the outer edges. A digital camera located above the maze captured images, which were transmitted to a personal computer running the Ethovision tracking software (see above). For each trial, the mouse was placed in the central square facing one of the open arms. It was allowed to explore freely for 5 min before being removed and returned to the home cage. Two measures were scored—the percentage of time spent in the open arms and the total distance traveled in the 5-min period.

#### Novel object recognition test

This test investigated the ability of the mouse to distinguish between known and unknown objects. The test was performed for 3 days. On day 1, the mouse was placed in an open field box for 10 min. During this time (habituation), the mouse became familiar with the empty arena. On day 2, two identical objects were placed in the arena, and the mouse was allowed to investigate these objects for 10 min. On day 3, the mouse was placed into the arena that now contained—in addition to one old, familiar object—a new object unknown to the mouse. To analyze the time spent by the mice to investigate the objects, i.e., the time spent very close to the objects, a circle of 2 cm to the object was drawn around the objects. The time spent by the mice close to the objects was determined by detection of the nose in the circle; it was then divided in bins of 2 min. The times spent to investigate the different objects were evaluated. The time spent with two identical objects (AA) or one familiar and one new object (AB) on two successive days (day 2 and day3) was measured. On day 1, no objects were presented. Data of female and male mice were pooled. The discrimination index was calculated as follows: (time spent with B − time spent with A) / (time spent with B + time spent with A) where 0 = equal exploration, < 0 = more time at object A, and > 0 = more time at object B. Discrimination index for WT and *Kcnh7*-KO mice was tested on day 2 and day 3.

#### Social interaction test

The social interaction test was performed in the open field arena. The apparatus was illuminated with 30 lux, and a digital camera was mounted above the setup. The videos were transmitted to a personal computer running Ethovision tracking software (s. above) equipped with three-point (nose, body center, and tail) detection settings. Two round cages (12 cm in diameter and 13 cm in height) with a heavy lid were placed in the arena. Active exploration was scored when the nose of the test mouse was detected within a radius of 2 cm around the metal cage. A mouse unknown to the test mouse was placed in one cage (stranger 1), whereas the second cage was left empty. After 10 min, a second unknown mouse (stranger 2) was placed in the previously empty cage, and the test mouse was allowed to explore for another 10 min. The time spent with stranger 1 compared to the object and the time spent with stranger 1 compared to stranger 2 was analyzed.

#### Pole test

This test investigates motor coordination. Mice were placed on top of a vertical rod (rough wood, 48.5 cm long, with diameter of 0.8 cm, illuminated with dim white light of 30 lux), grasping the rod with four paws and the head upright. The natural behavior of a mouse is to turn around 180° and to climb down along the pole. The ability to perform this behavior was evaluated. In case the mouse turned, it was recorded if it turned at the top (level 1, above 32 cm), at the middle (level 2, between 32 and 16 cm), or at the bottom of the rod (level 3, below 16 cm) and if it made use of its tail. To motivate the mouse to climb down, nesting material of the animals’ home cage was placed at the bottom of the pole. If a mouse did not start climbing down after 90 s, it was guided down by holding the nesting material in front of its nose, thereby leading it down. Each mouse had to perform three consecutive trials with an inter-trial interval (ITI) of 30 s.

#### Rotarod

The Ugo Basile accelerating rotarod test for mice was used (model: 47600; Comerio, VA, Italy). The testing area was illuminated diffusely with 30-lux dim white light. A digital camera captured videos that were transmitted to a computer running Ethovision tracking software (s. above). The test was performed in two steps. On day 1, the mice underwent five trials with ITI of 50 to 60 min. Trials 1 and 2 were performed at slow, constant speed (four rpm) for a maximum duration of 3 min. Trials 3 to 5 were performed with accelerating speed, from 4 up to 40 rpm within 4 min, with a maximum duration of 10 min. On day 2, three accelerating trials were carried out with a reduced ITI of 10 to 15 min. The performance of the mice was evaluated by scoring the latency to stay on the rotating rod. This task is an exquisite challenge and depends on the quality of motor coordination, strength of locomotor activity, and on the motivation to stay as long as possible on the rod.

#### Marble burying test

Two Typ III cages (26.7 cm by 42.7 cm) were filled with fine wooden bedding chips up to 5 cm and pressed tight. A 5 × 4 grid of 20 black glass marbles (16 mm in diameter) was placed evenly on top of the bedding. The cage was illuminated with dim white light of 20 lux. The mouse was placed in the cage, observed, and video-recorded for 30 min. The number of marbles buried was noted every 5 min. A marble was considered buried if more than two-thirds of it was covered with wooden chips. Between subjects, the bedding was stirred thoroughly, pressed down to have a plane surface and marbles were again placed on top. The marbles buried were counted manually in 5-min time bins.

### Statistics

Data were analyzed with GraphPad Prism 9.5 and IBM SPSS Statistics Version 28.0. Data were checked for normality with Shapiro-Wilk and Kolmogorov-Smirnov tests and, depending on the result, analyzed with parametric or nonparametric statistics. More factorial data, even if some datasets did not meet the assumption of normality, were analyzed with analysis of variance (ANOVA). All tests were performed two-sided. Data were initially checked for differences between males and females (three-way repeated measure ANOVA). If there were no significant effects of gender detected in the main parameters and since data for electrophysiology were also retrieved from both genders, data of males and females were combined for graphic presentation and further analysis. Data were checked for outliers with ROUT (Q = 1%). In the few cases outliers were detected, the values could be attributed to divergent individual behavior of the mice rather than to systemic errors; thus, they were not omitted. For correlational data the Pearson’s coefficient was calculated. Tests are indicated in the figure legends. Raw data, test of normality, and exact *P* values can be found in the source data. During the behavior experiments, the investigator was blind to the genotype of the subjects.

### Animal experiments

Experiments were carried out in accordance with German and European Union laws on the protection of experimental animals and following approval by the local authorities of the City of Hamburg (Committee for Lebensmittelsicherheit und Veterinärwesen, Authority of Soziales, Familie, Gesundheit und Verbraucherschutz Hamburg, Germany; TVA: Org 932, N083/2020). Animal experiments were performed according to the ARRIVE guidelines.
