# Supplementary material for: Purkinje cell hyperexcitability and depressive-like behavior in mice lacking erg3 (ether-à-go-go–related gene) K+ channel subunits
Source: Sci Adv. 2024 Oct 4;10(40):eadn6836. doi: 10.1126/sciadv.adn6836 (PMC11451553; doi:10.1126/sciadv.adn6836)
Supplement: Supplementary file 1 — Figs. S1 to S4 Tables S1 and S2 [file sciadv.adn6836_sm.pdf]

Science Advances
AAAS

Supplementary Materials for

Purkinje cell hyperexcitability and depressive-like behavior in mice lacking erg3 (ether-à-go-go–related gene) K^+ channel subunits

Jürgen R. Schwarz *et al.*

Corresponding author: Jürgen R. Schwarz, juergen.schwarz@zmnh.uni-hamburg.de;
Matthias Kneussel, matthias.kneussel@zmnh.uni-hamburg.de

Sci. Adv. **10**, eadn6836 (2024)
DOI: 10.1126/sciadv.adn6836

This PDF file includes:

Figs. S1 to S4
Tables S1 and S2

Suppl. Figure 1, related to Figure 5

Purkinje cells

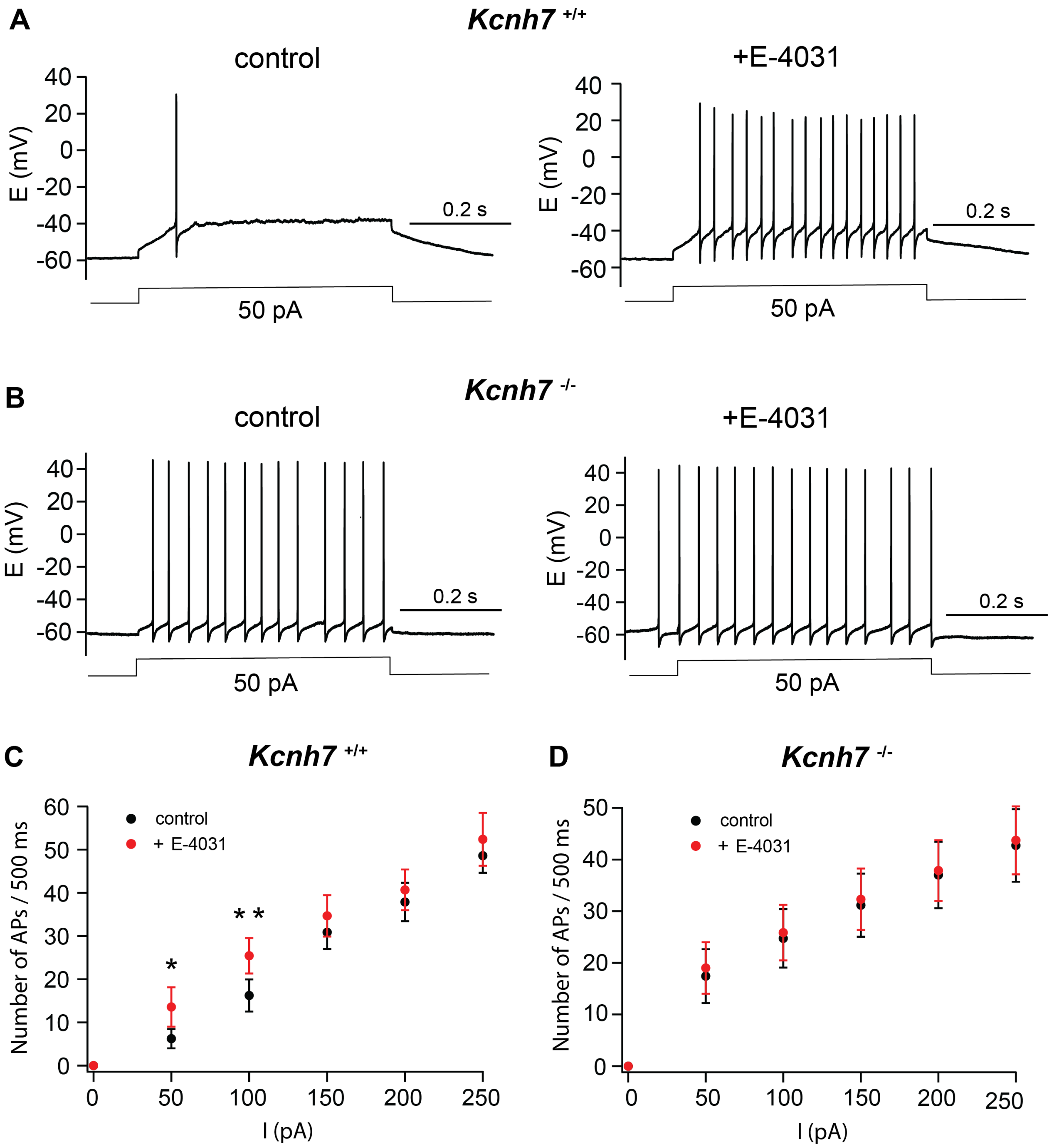

Figure S1 (related to Figure 5).

Application of E-4031 increases the frequency of repetitive activity in PCs of WT, but not of *Kcnh7*-KO mice.

Repetitive activity elicited with depolarizing current pulses. **A** Current-clamp recording from a PC of a WT mouse (age: 4 weeks). Left panel: a 50 pA rectangular depolarizing current pulse elicited a single action potential. Right panel: in the presence of E-4031 the membrane potential slightly depolarized and a series of action potentials without any accommodation was elicited. In addition, the time-to-peak of the first action potential was reduced. As a further indication for an increased excitability the threshold potential was significantly reduced by E-4031 (controls: 15.3±2.7 mV, +E-4031: 12.3±2.2 mV; n=11, p=0.0012). **B** repetitive activity elicited with a 50 pA depolarizing current pulse in a PC of a *Kcnh7*-KO mouse (age: 8 weeks) (left panel), which did not increase in its frequency in the presence of E-4031 (right panel). **C** Graphs plotting the number of action potentials elicited by 500 ms depolarizing current pulses of amplitudes increasing in steps of 50 pA. Left panel: WT-PCs (n=9, data from 6 mice). Right panel: PCs from *Kcnh7*-KO mice (n=11, data from 4 mice). No change in the threshold potential in PCs of *Kcnh7*-KO mice by E-4031 (controls: 12.9±2.2 mV, E-4031: 11.5±1.2 mV; n=13, p=0.33). The threshold potential in PCs of *Kcnh7*-KO mice was similarly low as in control WT neurons in the presence of E-4031. The increase in repetitive firing was supported by the increase in the membrane resistance by E-4031 (controls: 95.1±7.2MΩ, + E-4031: 107.2±6.7MΩ; n=12, p=0.002). The membrane resistance was determined from the change in membrane potential upon a 100pA hyperpolarizing current injection. The membrane resistance of PCs of *Kcnh7*-KO mice was not changed by E-4031 (controls: 131.1±9.1 MΩ, + E-4031: 137.6±12.6 MΩ, n=13, p=0.49).

Suppl. Figure 2, related to Figure 5

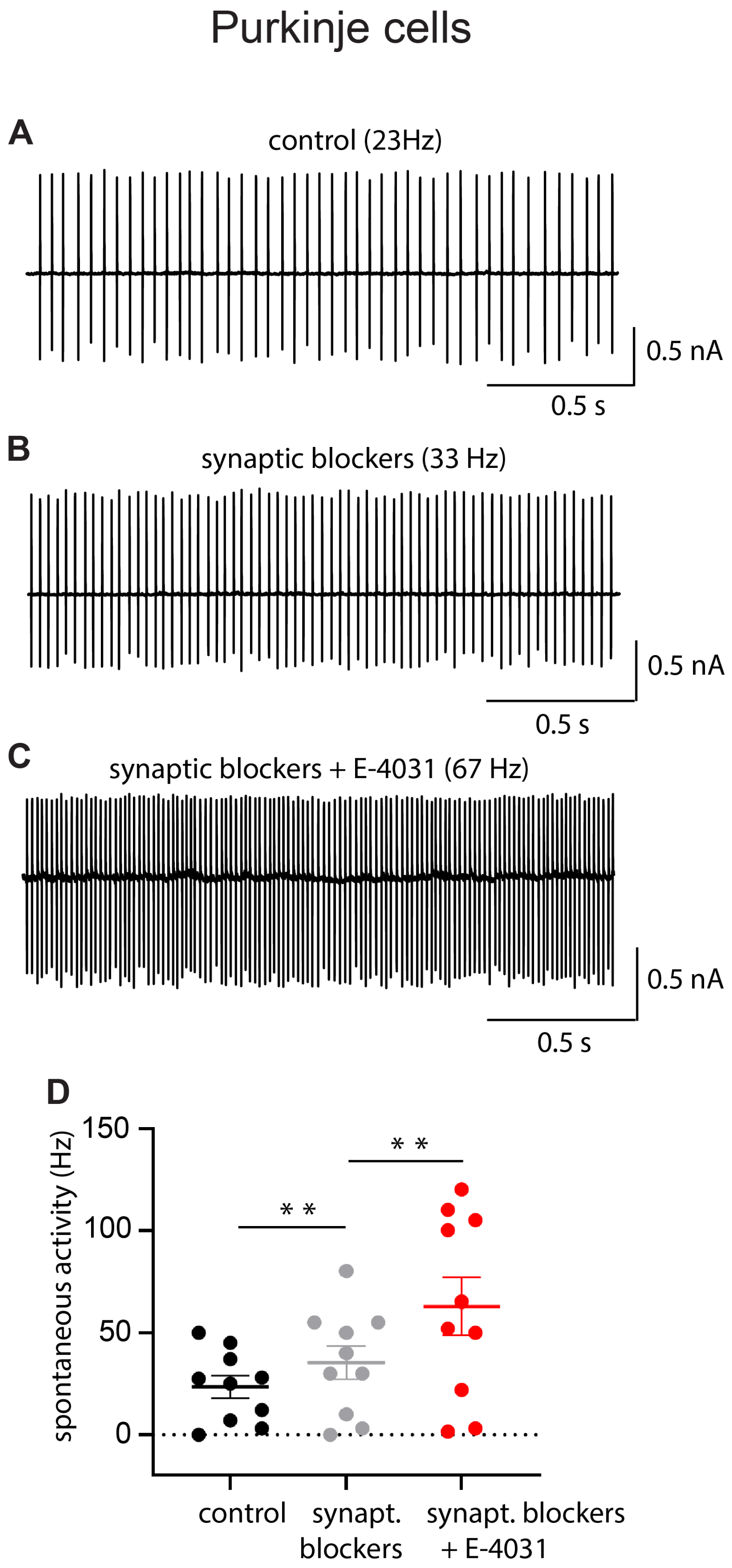

Figure S2 (related to Figure 5).

The frequency of spontaneous activity increased in the presence of synaptic blockers and further increased upon the additional application of E-4031.

A-C On-cell recordings from a PC of a WT mouse: control (**A**), in the presence of synaptic blockers (**B**), and in the presence of synaptic blockers + 10 μM E-4031 (**C**). **D** Graph plotting the frequency of spontaneous activity of WT-PCs (n=10, data from 8 mice aged 4-7 weeks). Significant increase of spontaneous activity in the presence of synaptic blockers as compared to the activity of control recordings (control: 23.5±5.5 Hz; in the presence of synaptic blockers: 35.3±8.2 Hz; p=0.006). Application of 10 μM E-4031 led to an additional significant increase of firing activity (62.9±14.1 Hz, p=0.006).

Suppl. Figure 3, related to Figure 7

CA1 neurons

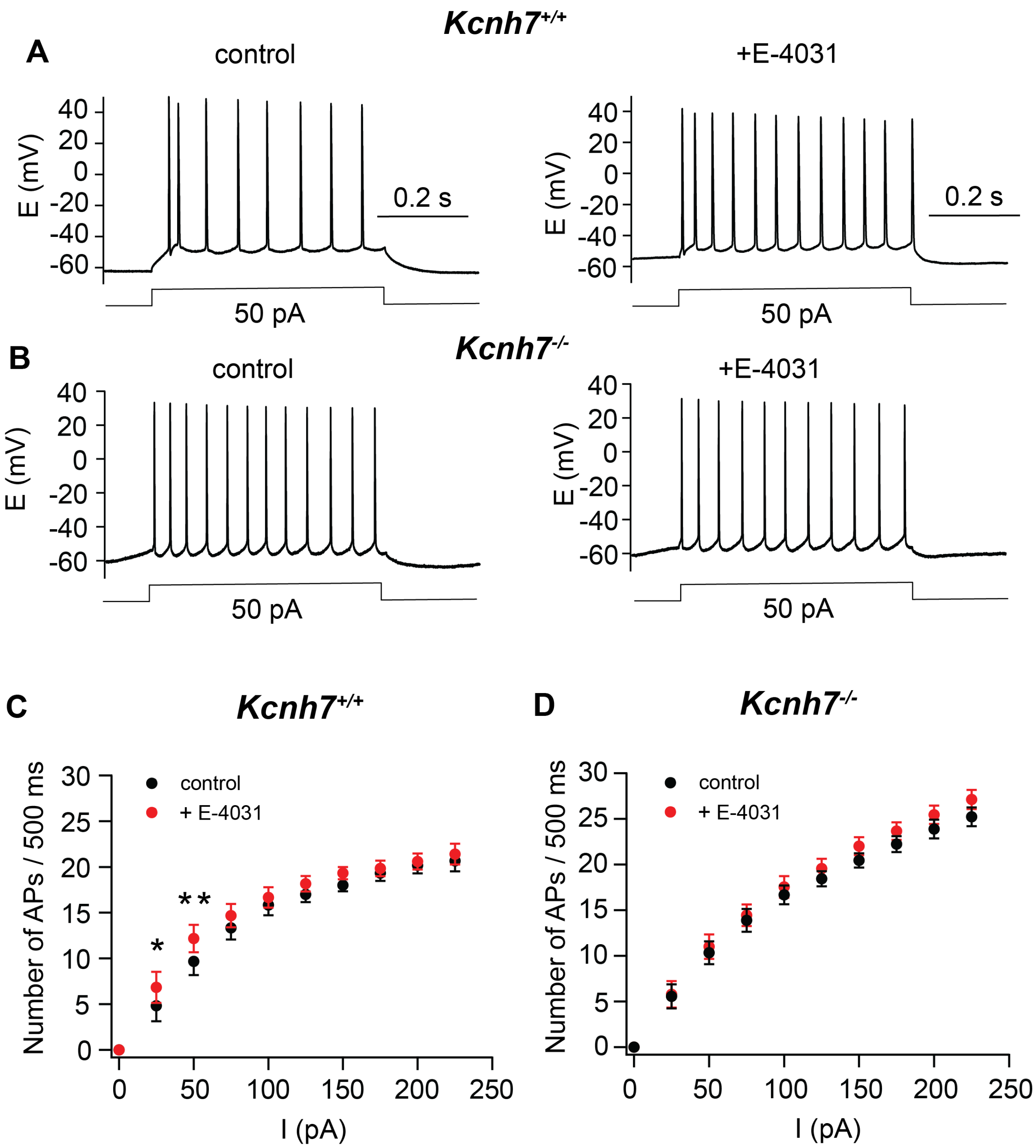

Figure S3 (related to Figure 7).

Application of E-4031 increases repetitive activity in WT - CA1 neurons but not in CA1 neurons of *Kcnh7*-KO mice.

Repetitive activity elicited with depolarizing current pulses in steps of 25 pA. **A** In a WT-CA1 neuron a 50pA depolarizing current pulse elicited 8 action potentials (left panel), in the presence of E-4031 the number of action potentials increased (right panel). **B** Repetitive activity (left panel) of a CA1 neuron of a *Kcnh7*-KO mouse elicited with a 50pA depolarizing current pulse which did not change in the presence of E-4031 (right panel). **C** Graph plotting number of action potentials elicited by 500 ms increasing depolarizing current pulses in steps of 25 pA in CA1 neurons of WT (n=6, data from 3 mice, aged 2 weeks) and *Kcnh7*-KO mice (n=9, data from 2 mice, aged 2 weeks). In WT-CA1 neurons (same neurons as above) the threshold potential was significantly reduced in the presence of E-4031 (control: 11.4±0.7 mV, +E-4031: 8.8±0.7 mV, p=0.002, n=6). In CA1 neurons of *Kcnh7*-KO mice (same neurons as above) the threshold remained high and was not influenced by E-4031 (12.6±1.5 mV, +E-4031: 13.2±1.5, p=0.13, n=9).

Suppl. Figure 4, related to Figure 7

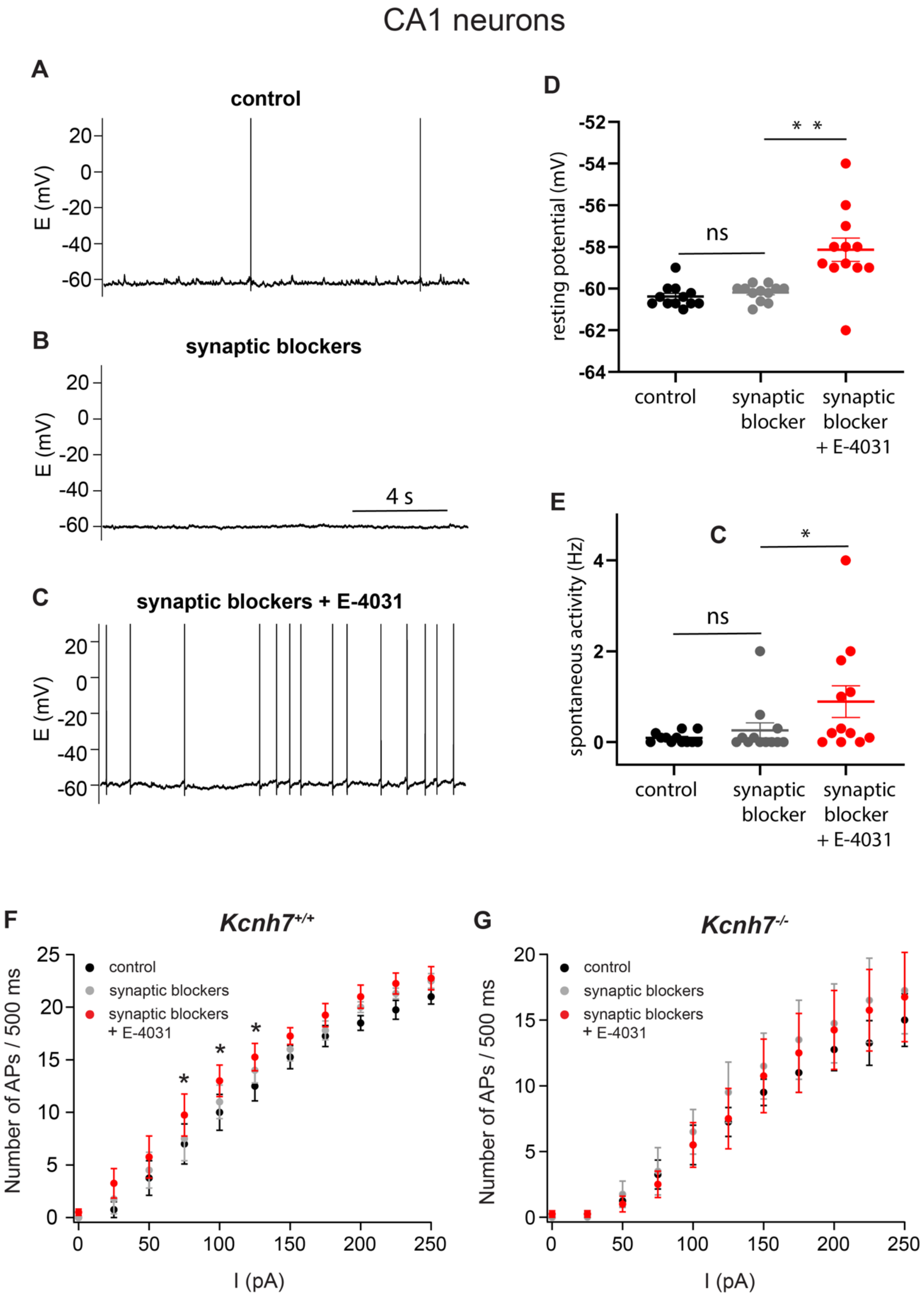

Figure S4 (related to Figure 7).

Synaptic blockers do neither change neuronal excitability of CA1 neurons from WT mice nor that of CA1 neurons of *Kcnh7*-KO mice

Current clamp recording of spontaneous activity of a WT-CA1 neuron in the absence (**A**) and presence (**B**) of synaptic blockers, as well as in the presence of synaptic blockers + E-4031 **(C). D** Graph plotting the resting potential (Er) of CA1 neurons (n=12, data from 5 mice aged 5-8 weeks) in the absence (Er: -60.4±0.2 mV) and presence of synaptic blockers (-60.2±0.1 mV, p = 0.14) and in the additional presence of E-4031 (-58.1± 0.6 mV, p = 0.0037). **E** The frequency of spontaneous activity did not change in the presence of synaptic blockers, whereas in the presence of 10 µM E-4031 spontaneous activity increased significantly (control: 0.09±0.03 Hz, synaptic blocker: 0.26±0.17 Hz, p=0.32; synaptic blockers + 10 µM E-4031: 0.89±0.35 Hz, p=0.028). **F, G** Repetitive activity recorded by 500 ms depolarizing current pulses increasing in steps of 25 pA in WT-CA1 neurons (n= 4, data from 2 mice, aged 5 weeks) and in CA1 neurons from Kcnh7-KO mice (n= 4, data from 3 mice, aged 6 weeks). There was no change in repetitive activity upon application of synaptic blockers. In contrast, there was a significant increase in repetitive activity in the presence of E-4031 in WT-CA1 neurons.

Supplementary Table 1. Time constants of erg current deactivation determined in Purkinje cells of WT and *Kcnh7*-KO mice, CA1 neurons and HEK293 cells expressing erg1a or erg3 channels.

	-80 mV<br>mean ± SEM	-100 mV<br>mean ± SEM	-120 mV<br>mean ± SEM
PC WT	100.0 ± 12.9 ms (n=10)	43.9 ± 4.7 ms (n=12)	23.1 ± 2.1 ms (n=7)
CA1 WT	41.6 ± 6.1 ms (n=8)	23.2 ± 3.2 ms (n=10)	16.0 ± 1.8 ms (n=16)
erg 3	50.1 ± 2.6 ms (n=18)	25.0 ± 1.4 ms (n=18)	17.4 ± 0.9 ms (n=18)
PC *Kcnh7*-KO	152.8 ± 34.7 ms (n=5)	63.3 ± 5.0 ms (n=6)	32.0 ± 3.0 ms (n=8)
erg 1a	197.3 ± 15.0 ms (n=12)	63.5 ± 3.9 ms (n=12)	26.8 ± 1.3 ms (n=12)
Cond. *Kcnh2*-KO	29.8 ± 2.2 ms (n=8)	16.7 ± 1.4 ms (n=9)	

Time constants of erg current deactivation determined in erg currents measured in PCs, CA1 neurons, and HEK293 cells expressing either erg1a or erg3 channels. Wild-type PCs (PC WT), wild-type CA1 hippocampal neurons (CA1 WT), PCs of *Kcnh7*-KO mice (PC *Kcnh7*-KO), PCs from conditional *Kcnh2*-KO mice (Cond. *Kcnh2*-KO).

Supplementary Table 2

Change of resting potential (Er) in the presence of 10 µM XE991

Purkinje cells
No Er change in Purkinje cells of WT mice
Control: -58.0±1.5 mV, + 10 µM XE991: -58.0±1.5 mV, n=3

WT CA1 neurons
Depolarization of 4.0 ± 0.9 mV.
Control: -60.0 ± 0.3 mV, + 10 µM XE991: -56.3±0.9 mV, n=7
Er difference: 4.0 ± 0.9 mV

CA1 neurons of Kcnh7-KO mice
Depolarization of 5.6 ± 0.9 mV.
Control: -59.6 ± 0.4 mV, + XE991: -53.8±0.8 mV, n=8
Er difference: 5.6 ± 0.9 mV

No significant difference between XE991-induced Er change of CA1 neurons of WT and *Kcnh7*-KO mice (p = 0.24).
